# Unlocking the Bioactivity of Cyperi Rhizoma as a Functional Food: Insights From Culinary Processing and Herb Combinations

**DOI:** 10.1002/fsn3.71808

**Published:** 2026-05-15

**Authors:** Yuehan Liu, Liyuan Xu, Liting Lin, Maoyuan Jiang, Wan Liao, Tianhui Gao

**Affiliations:** ^1^ School of Pharmacy Qilu Medical University Zibo Shandong China; ^2^ School of Pharmacy Chengdu University of Traditional Chinese Medicine Chengdu Sichuan China; ^3^ Key Laboratory of Standardization of Chinese Medicine (Chengdu University of Traditional Chinese Medicine), Ministry of Education Chengdu Sichuan China; ^4^ Lab for Innovation & Effective Uses of Chinese Drug Germplasm Resources Chengdu Sichuan China

**Keywords:** agricultural byproduct upcycling, biological activity mechanism, Cyperi Rhizoma, herb‐food synergy

## Abstract

Cyperi Rhizoma (CR), a globally invasive weed threatening major crops, paradoxically represents an underutilized resource for sustainable nutraceutical development. In order to transform this agricultural pest into a viable functional food ingredient, this review explores how culinary‐inspired processing (vinegar, wine, salt, etc.) and food‐compatible botanical pairings synergistically enhance the bioactivity of CR. We demonstrate that traditional processing deglycosylates flavonoids and modulates volatile oils, boosting bioavailability and analgesic, antidepressant efficacy validated in vivo. Herb pairs with food‐medicines generate novel metabolites and amplify bioactivity, as exemplified by the CR‐Alpiniae Officinarum Rhizoma herb pair that promotes gastric ulcer inhibition via gut barrier repair. Moreover, CR‐based formulations show clinical success in digestive/neurological disorders. This work connects weed control strategies with value‐added food uses, including spice blends and nanoparticle‐encapsulated supplements, establishing the scientific basis for transforming CR into scalable, culturally adapted functional foods. These innovations target global health needs while reducing agricultural damage caused by this invasive plant.

## Graphical abstract

Potential pharmacological activities of *Cyperus rotundus* L. (CR) when paired with six medicinal and edible herbs, exhibiting beneficial effects in anti‑inflammation, anti‑depression, pain relief, and so on.

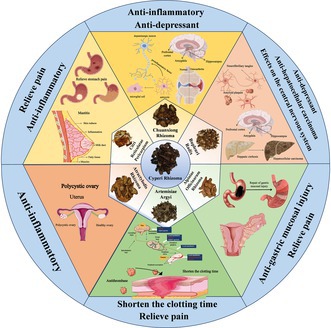

## 1 Introduction

**FIGURE 1:**
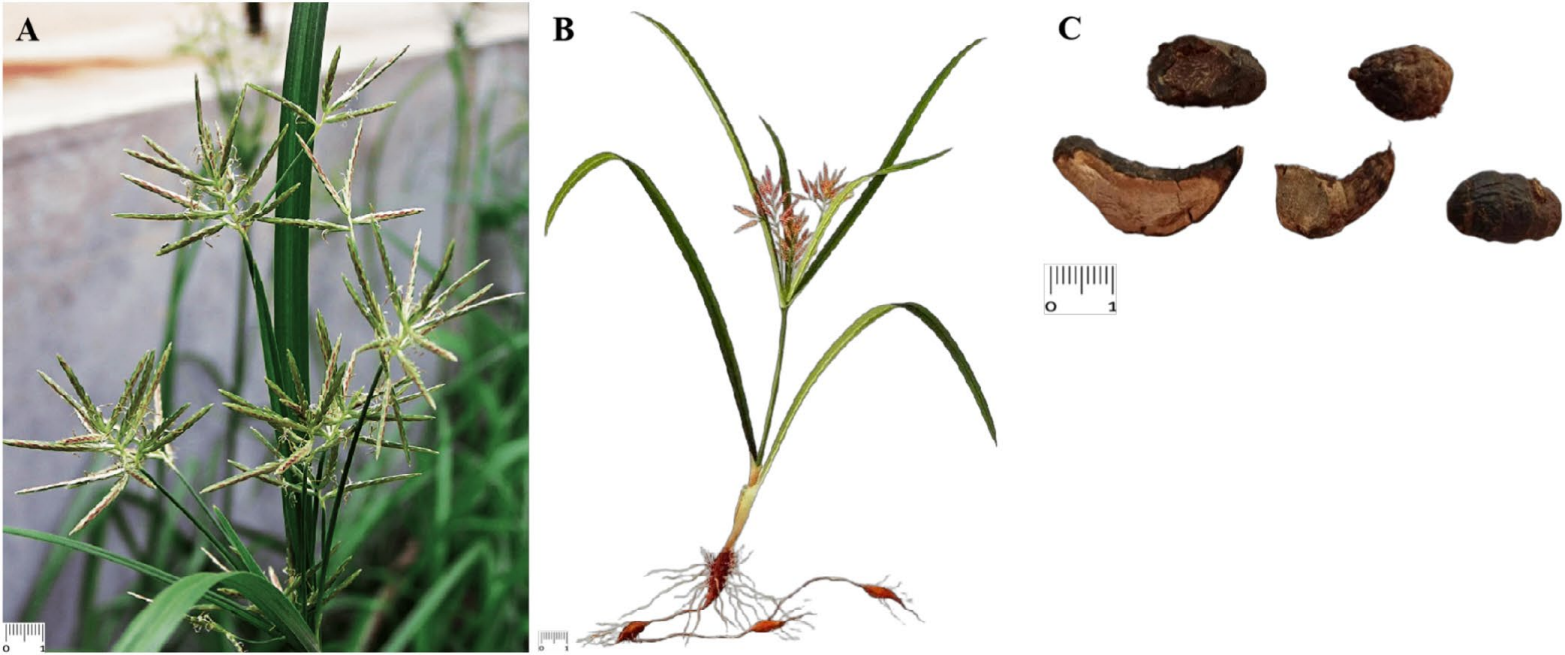
Schematic diagram of CR plant. (A) The wild growing state of CR plant, (B) Hand‐painted whole‐plant image, (C) CR in the form of TCM decoction pieces.

Cyperi rhizoma (CR) is the dried rhizome of *Cyperus rotundus* L., a plant in the family Cyperaceae (Figure 1), known as Xiangfu in Traditional Chinese Medicine (TCM), is a pervasive perennial weed infesting major crops such as maize, sugarcane, and vegetables worldwide. Its robust rhizome system facilitates rapid colonization, reducing yields by 20%–90% across diverse agroecosystems. For instance, densities of 200–500 plants/m^2^ cause 32%–79% losses in tomatoes, sweet peppers, and maize, while sugarcane productivity declines by 45.2% under high infestation (Morales‐Payan et al. 1997, 1998; Ye 2008). This resilience and competitiveness rank it among the world's top ten invasive weeds (C. Wang 2020). Paradoxically, beyond its agricultural impact, CR has been valued for millennia as a medicinal and food resource. Documented in Chinese *Ming Yi Bie Lu* (220–450 AD) as a “gynecological essential”, it also features in pharmacopeias of India, Iran, Japan, and Bangladesh, where rhizomes treat ailments ranging from diarrhea and fever to digestive disorders and lactation support. Its tubers are edible despite bitterness and nutritionally valued in India (Rani and Padmakumari 2012). In Tunisia, its tubers could alleviate flatulence, and Nigerian industries extract its starch for food and confectionery (Umerie and Ezeuzo 2000). Traditional Thai use for gastrointestinal ailments containing dyspepsia and diarrhea further highlights its dual role in functional food development (Neamsuvan and Ruangrit 2017). Furthermore, CR also finds applications in perfumery, spice formulations, and nutraceutical products, exhibiting considerable international demand in Arab nations, Africa, China, India, and other territories. According to ISO/TR 23975, CR is ranked 82nd in the comprehensive ranking of all herbals and 51st in terms of frequency of use in pharmacopeias and local standardized formulas in various countries. During the period spanning from 2017 to 2021, the international trade of single herbal medicines between China and Japan was recorded to be valued at 256,0798 kg, placing it in the 69th position in terms of trade volume. This reflects the fact that CR is widely used in many countries and regions.

Modern studies validate CR's pharmacological potential, linking its bioactive compounds—including terpenoids (α‐cyperone, nootkatone), flavonoids, and phenolic acids—to antidepressant, anti‐inflammatory, antimicrobial, and antioxidant effects. Its essential oil exhibits potent antibacterial activity against *Staphylococcus aureus* with the MIC of 10 mg/mL (Zhang et al. 2017), and its ethyl acetate and methanol extracts from leaves and flowers have antimutagenic activity against Aflatoxin B1 (Kilani et al. 2005). Despite its agricultural notoriety, these properties position CR as a resource for functional foods and therapeutics. Critically, traditional processing methods with food could enhance its efficacy, such as vinegar‐processing, which augments its “qi‐regulating” properties in TCM (Chen et al. 2022), while combining CR with food‐medicines like Alpiniae officinarum rhizome (Gaoliangjiang in Chinese, GLJ) synergistically alleviates gastric ulcers (Qu et al. 2021). However, while extensive research exists on isolated CR and its extracts, no comprehensive review addresses how food‐compatible processing or synergistic pairing with dietary botanicals amplifies its bioactivity, which is a critical gap given its potential in nutraceutical development.

**FIGURE 2:**
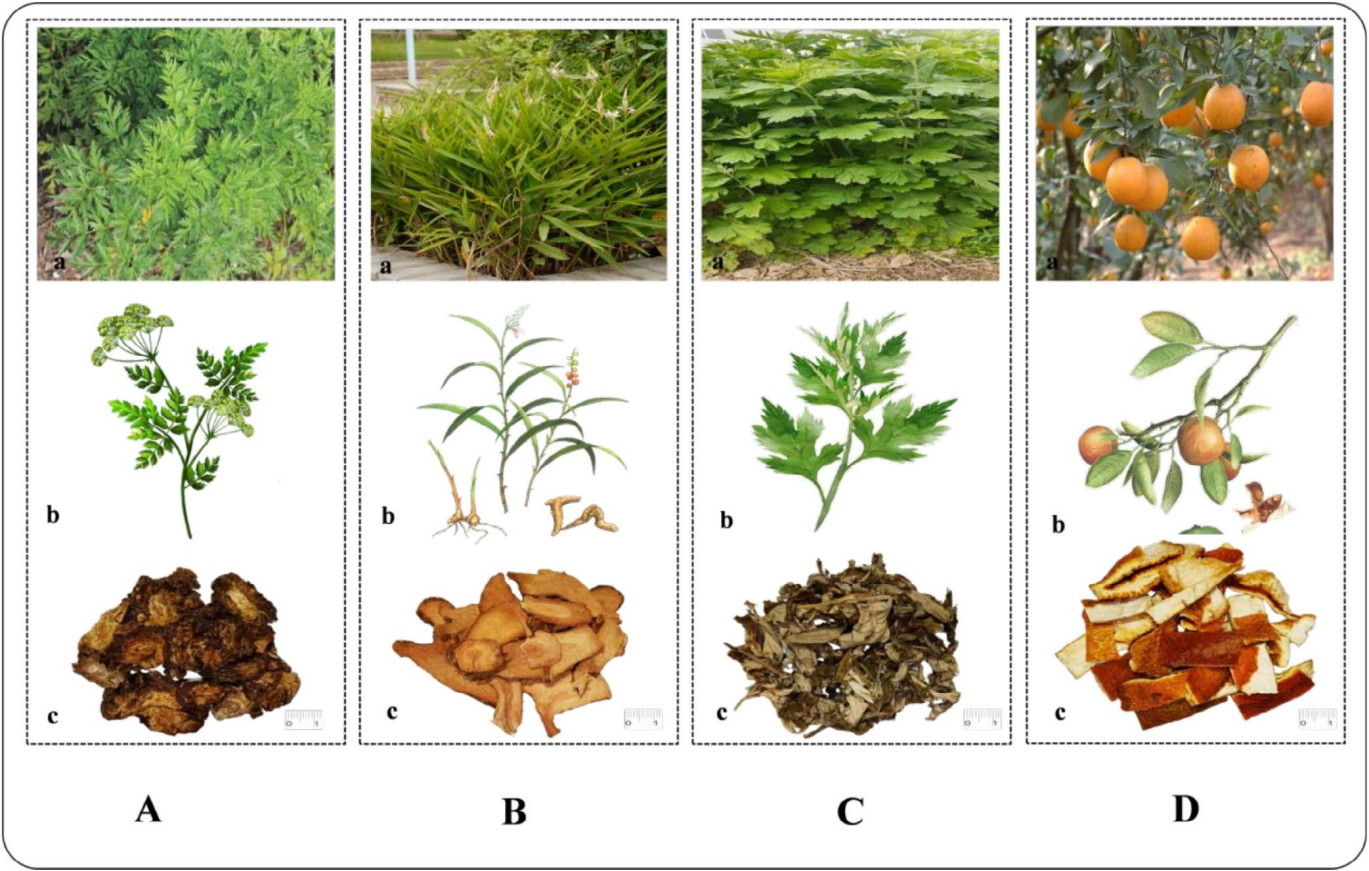
CX (A); GLJ (B); AY (C); CP (D); Schematic diagram of herb plant. (a) The wild growing state of herb plant. (b) Hand‐painted whole‐plant image; (c) herb in the form of TCM decoction pieces.

**FIGURE 3:**
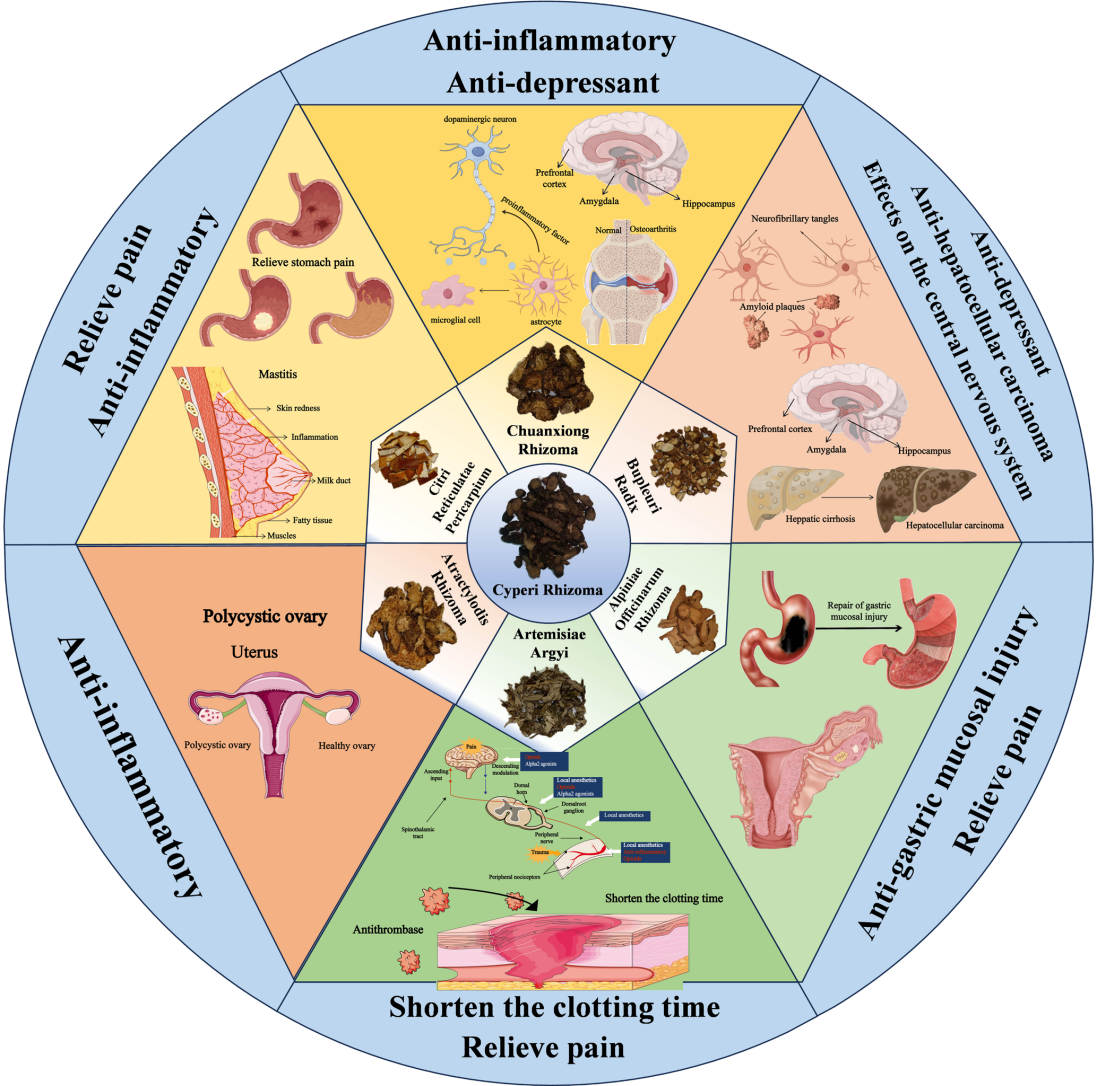
Schematic diagram of potential pharmacological activities of various herb pairs containing CR.

This review synthesizes current data on food‐driven synergies enhancing the bioactivity of CR. The search terms “Cyperi rhizoma, *Cyperus rotundus* L., Xiangfu, herb‐pair, processing, phytochemistry and pharmacology” were used to obtain the literatures from electronic databases such as PubMed, Web of Science, ScienceDirect, China National Knowledge Infrastructure (CNKI), Food and Health Information (IFIS), and International Food Information Service (FSTA). The information provided in this review is to illustrate the chemical interactions, pharmacological effects and clinical applications of CR after processing with various foods such as vinegar, wine, salt and rice, as well as the combination of CR with food‐compatible botanicals such as GLJ, Citri reticulatae pericarpium (Chenpi in Chinese, CP), Artemisiae argyi folium (Aiye in Chinese, AY), Chuanxiong Rhizoma (Chuanxiong in Chinese, CX; Figure 2). Elucidating the mechanisms underlying these synergies may enable repurposing this agricultural pest into a sustainable resource for nutraceuticals, functional foods, and herbal formulations (Figure 3). This approach bridges weed management objectives with value‐added applications in global food and health systems.

## 2 Culinary‐Inspired Processing of CR

The processing of TCM shares remarkable similarities with culinary techniques, as both require the addition of specific auxiliary materials and undergo specialized procedures to significantly enhance their efficacy. The processing technique, called PaoZhi in Chinese, is regarded as a core technology of TCM and a unique traditional skill exclusive to Chinese medicine. It has been recognized as one of Chinese important national‐level intangible cultural heritages. The processing history of CR dates back to ancient times, with its earliest documentation found in *Lei Gong Pao Zhi Lun* during the Northern and Southern Dynasties period (AD 420–479). Over time, more than 20 distinct processing methods have been developed (Chen et al. 2023). Statistical analysis shows that the most frequently used food‐based auxiliary materials include: yellow rice wine, salt, vinegar, fresh ginger, rice, milk, Foeniculi Fructus, black soybean, brown sugar, processed honey, Alpiniae Oxyphyllae Fructus, and white radish (Li, Li, et al. 2025). Based on the different food‐based auxiliary materials and processing methods, CR processed products can be categorized into: vinegar‐processed, wine‐processed, ginger‐processed, salt‐processed, double‐processed, quadruple‐processed, and quintuple‐processed (Table 1).

**TABLE 1:** The records of processing method of CR.

Processing method	Food‐based auxiliary materials	Sensory description	Source
Vinegar‐processed	Rice vinegar	Externally blackish‐brown; with a slight vinegar aroma; tastes slightly bitter	Pharmacopeia of the People's Republic of China (2025)
Wine‐processed	Yellow rice wine	Externally purplish‐red with scorch marks; aromatic	Processing Standards for Chinese Herbal Decoction Pieces of: Gansu Province (1980), Henan Province (2005), Hunan Province (2010), Shandong Province (2012), Sichuan Province (2015), Chongqing Municipality (2006)
Ginger‐processed	Fresh ginger juice	Externally brownish‐brown or blackish‐brown; internally yellowish‐white or reddish‐brown with a distinct cambium ring; tastes slightly bitter with a tinge of pungency and hotness	Processing Standards for Chinese Herbal Decoction Pieces of: Sichuan Province (2015), Chongqing Municipality (2006)
Salt‐processed	Brine	Externally brownish‐brown or blackish‐brown; internally yellowish‐white or reddish‐brown with a distinct endodermis ring; tastes slightly bitter and salty	
Double‐processed	Rice vinegar, yellow rice wine/Chinese Baijiu	The entire substance appears blackish; longitudinal wrinkles are visible on the surface, and some samples may show residual fibrous root scars and transverse rings; texture hard; fractured surface dark brown and lustrous; odor faint; taste slightly bitter	Standards for Chinese Herbal Decoction Pieces of: Yunnan Province (2005), Shanghai Municipality (2018)
Quadruple‐processed	Rice vinegar, yellow rice wine/Chinese Baijiu, salt, ginger	Externally brownish‐brown or blackish‐brown; cut surface yellowish‐brown to dark brown; aromatic; tastes slightly bitter and salty	Processing Standards for Chinese Herbal Decoction Pieces of: Fujian Province (2012), Guangdong Province (1984), Guangxi (2007), Henan Province (2005), Sichuan Province (2015), Chongqing Municipality (2006), Hunan Province (2010), Jiangxi Province (2008)
	Rice vinegar, yellow rice wine, salt, brown sugar/processed honey	Externally dark brown or blackish‐brown, with a horn‐like texture; fractured surface yellowish‐brown; tastes slightly salty, sour, and pungent; with a delicate, refreshing aroma	Processing Standards for Chinese Herbal Decoction Pieces of: Yunnan Province (2005), Guizhou Province (2005), Hubei Province (2018), Ningxia (2017)

### 2.1 Processing‐Induced Change in Constituents

During the processing of CR, variations in auxiliary materials or temperature parameters may induce changes in either the chemical composition profile or quantitative content of specific constituents within the medicinal material. The volatile oils in CR are recognized as the primary active constituents, predominantly comprising alkenes, alkanes, alcohols, and ketones (Yang et al. 2021). The results demonstrated that vinegar‐processed CR exhibited reduced total volatile oil content, with decreased levels of α‐cyperone and nootkatone, while cyperotundone content increased (Ji et al. 2015). Qiao et al. found that wine‐processing and quadruple‐processing methods elevated α‐cyperone but reduced cyperotundone, revealing a negative correlation between these two markers (Qiao et al. 2022). Li's further research indicated hierarchical α‐cyperone concentrations: quintuple‐processed (0.90 ± 0.03 mg/mL) > quadruple‐processed (0.64 ± 0.03 mg/mL) > vinegar‐processed (0.34 ± 0.02 mg/mL; Li, Lu, et al. 2018). Additionally, studies showed that quadruple‐processing increased volatile oil yield and introduced over 20 additional components compared to crude material (Liang et al. 2018). It should be noted that conflicting results have been reported regarding changes in α‐cyperone content following vinegar processing. This discrepancy may be attributed to variations in processing parameters, including vinegar concentration, soaking duration, and frying temperature, which are not consistently standardized across studies. Additionally, differences in analytical methods such as extraction solvents and detection wavelengths could contribute to the inconsistent findings. These observations highlight the critical need for establishing standardized processing protocols to ensure reproducibility and comparability of results.

**FIGURE 4:**
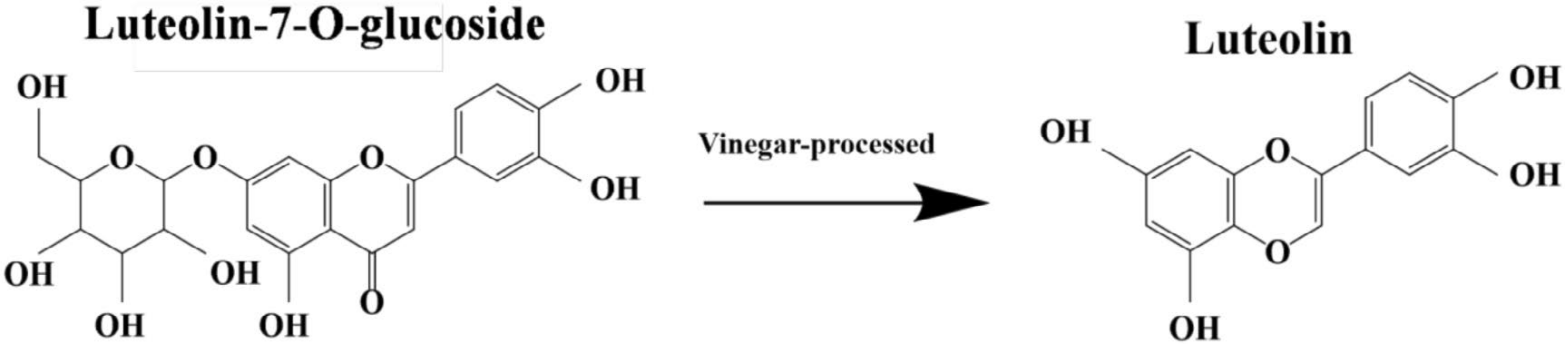
Transformation pathway of Luteolin‐7‐O‐glucoside in vinegar processing.

The flavonoid components in CR were also altered during processing. Researchers found that the contents of rutin and luteoloside significantly decreased after vinegar processing, while luteolin levels increased significantly (*p* < 0.05) (Song 2021). This was likely due to the hydrolysis of Luteolin‐7‐O‐glucoside during vinegar processing, leading to Luteolin‐7‐O‐glucoside deglycosylation and conversion into luteolin (Figure 4). Additionally, another study discovered that in the quadruple‐processing of CR, the amounts of salt and vinegar used, as well as the frying temperature, were positively correlated with its total flavonoid (Hu et al. 2012) Additionally, Li et al. determined through perchloric acid colorimetry that the total saponin increased by 28.21% in vinegar‐processed and 22.48% in wine‐processed CR compared to crude CR. These results demonstrated that processing significantly elevated saponin levels, with vinegar‐processing yielding higher saponin content than wine‐processing (Li et al. 2010).

### 2.2 Processing‐Induced Change in Pharmacodynamics

#### 2.2.1 Analgesic Effect

Four different processed products of CR were evaluated for analgesic efficacy using the hot‐plate test. The quadruple‐processed CR demonstrated the strongest analgesic effect, followed by vinegar‐processed, then crude CR, while wine‐processed CR reduced analgesic activity (Guo, Wang, et al. 2017; Guo, Dong, et al. 2017). The quadruple‐processed form also exhibited significant anti‐dysmenorrheal properties. Zhao et al. identified the petroleum ether fraction as the key active fraction for dysmenorrhea relief, containing stigmasterol, daucosterol, physcion, and palmitic acid (Zhao et al. 2018). Further studies revealed synergistic intestinal absorption between cyperotundone and α‐cyperone, with other components in the petroleum ether fraction enhancing their bioavailability (Hu et al. 2021). Guo et al. confirmed that α‐cyperone, cyperotundone, and sugeonol are 3 active anti‐dysmenorrheal constituents in quadruple‐processed CR, likely acting through synergistic mechanisms (Guo, Wang, et al. 2017; Guo, Dong, et al. 2017).

Li et al. demonstrated enhanced analgesic effects of vinegar‐processed CR through behavioral pain assessments in rats, showing significant reductions in leg retraction duration and paw licking time. The medicated serum from vinegar‐processed CR markedly decreased c‐Fos protein expression, suppressed Fos‐like immunoreactive neuron activity in spinal dorsal horns (associated with pain signal transmission), and blocked nociceptive signaling at the spinal level, thereby potentiating analgesia (Li and Hu 2013). Sun et al. further revealed that compared to crude CR, the aqueous extract of vinegar‐processed CR exhibited stronger uterine contraction inhibition in rats, reducing myometrial tension with faster onset and prolonged duration, indicating superior anti‐dysmenorrheal efficacy (Sun et al. 2007).

#### 2.2.2 Antidepressant Effect

Vinegar‐processed CR demonstrated enhanced antidepressant effects by promoting body weight gain in rats, resolving ecchymosis, increasing spontaneous activity, improving behavioral flexibility, and reducing blood viscosity (Sheng et al. 2016). Studies showed that the volatile oil of vinegar‐processed CR significantly reduced immobility time in the tail suspension test and forced swim test in mouse models (Chen et al. 2023). Liu et al. suggested that the antidepressant mechanism involved elevated 5‐HT levels in the brain. The primary active components responsible for the antidepressant effects were identified as α‐cyperone, isolongifolen‐5‐one, caryophyllene oxide, Ledene oxide‐(II), and eudesmol in vinegar‐processed CR (Liu et al. 2020).

#### 2.2.3 Other Pharmacological Effects

Zhou et al. found that the ethyl acetate fraction, n‐butanol fraction, and aqueous fraction of vinegar‐processed CR significantly improved gastrointestinal function in rats, as evidenced by a marked reduction in gastric residual rate and increased small intestinal propulsion rate (*p* < 0.05). Vinegar‐processed CR also significantly elevated plasma levels of motilin and gastrin in rats (*p* < 0.05), which were likely attributed to its active constituents promoting gastrointestinal motility (Zhou et al. 2016).

In a separate study, Guo et al. investigated the anti‐inflammatory effects of 4 differently processed CR preparations using a xylene‐induced mouse ear edema model. Results demonstrated that all preparations inhibited xylene‐induced ear swelling, with efficacy ranked as follows: vinegar‐processed > quadruple‐processed > crude > wine‐processed. This suggested that processing CR with culinary adjuvants including vinegar, wine, salt or ginger enhanced its anti‐inflammatory properties (Guo, Wang, et al. 2017; Guo, Dong, et al. 2017).

## 3 Food‐Compatible Synergistic Pairings of CR

### 3.1 CR‐CX Herb Pair

CX is the dried rhizome of *Ligusticum chuanxiong* Hort. As a representation of promoting blood circulation and removing blood stasis, CX is warm, pungent, and bitter, and it stimulates blood circulation (Figure 2A). CX is used to treat chest pain, menstrual disorders, dysmenorrhea, headache, and rheumatism. The main active ingredients of CX are phthalates, terpenes, polysaccharides, etc. CX is mainly used in clinical practice in the cardiovascular, nervous, and respiratory systems (Jin et al. 2013). CX is a genuine regional medicinal material (Daodi herb) primarily produced in Sichuan Province. Approximately 90% of commercially available CX originates from Sichuan cultivation bases (Jia 2007), notably Pengzhou, Dujiangyan, and Meishan. Local cultivation has achieved significant industrial scale. Production employs an eco‐friendly cultivation model characterized by: a paddy‐upland rotation system alternating Ligustici Rhizoma with rice (Tang et al. 2020). CX, approved for use in health foods in China, has demonstrated specific functional applications according to market research conducted over the past decade. The primary health benefits claimed for CX‐containing products are enhancing immunity, improving sleep, and assisting in blood lipid regulation (Li et al. 2024). Furthermore, CX serves as a natural additive in cosmetic formulations, exhibiting skin‐hydrating, skin‐whitening, and spot‐eliminating effects (Zhang 2014).

CR‐CX herb pair is one of the most representative formulas, and it has been demonstrated to enhance the efficacy of promoting the flow of qi in the liver and the treatment of emotional constraint. For example, the classic Yue Ju Pill (YJP) and Chai Hu Shu Gan powder (CHSGP) contain CR‐CX herb pair. Both of them can be widely employed in the treatment of depression (Deng et al. 2011), polycystic ovary syndrome (Zhang 2004), and so on. It also demonstrates extensive applications in health supplements and food products. For instance, the Chinese patent application “A Chinese Veterinary Medicine Oral Solution for the Prevention and Treatment of Poultry Liver Disease and Its Application” (Application date: August 22, 2023; Published application number: CN115068546B) utilizes a formulation containing herbs such as Artemisiae Scopariae Herba (Yinchen), Plantaginis Semen (Cheqianzi); “A Chinese Medicinal Composition with Antioxidant Properties and Method for Preparing a Health Food Product Therefrom” (Application Date: October 26, 2018; Publication application number: CN108704049A) utilizes a formulation containing herbs such as Lonicerae Japonicae Flos (Jinyinhua), Perillae Folium (Zisu); and “Traditional Chinese Medicinal Composition and Traditional Chinese Medicinal Wellness Tea, and Methods for Their Preparation and Uses” (Application Date: June 8, 2018; Publication application number: CN108126160A) utilizes a formulation containing herbs such as Galli Giaerii Endothelium Corneum (Jineijin), Mume Flos (Lvmeihua).

#### 3.1.1 Bioactive Components Variation of the CR‐CX Herb Pair

Zhang et al. employed HPLC to ascertain the alterations in 70% methanol extraction of CR before and after combining with CX. The findings revealed that the concentration of ligustrazine, ferulic acid, and ligustilide in CX increased following its compounding with CR (Zhang, Feng, et al. 2011; Zhang, Xu, and Wang 2011). Furthermore, the relative dissolution rates of ligustilide in the 50% and 95% ethanol extraction of the CR‐CX herb pair were superior to those extracted from CX alone (1724 μg/g with 50% ethanol and 1748 μg/g with 95% ethanol). The pairing of CR and CX in varying ratios results in disparate effects on the chemical composition. When the ratio of the CR‐CX herb pair is 1:1 or 2:1, the dissolution rate of α‐cyperone and nootkatone ethanol/water mixture can be increased to 59.51 μg/g and 82.00 μg/g. Based on the predicted results from response surface analysis, a combination of CR with CX at a ratio of nearly 1:1 resulted in the maximum dissolution of volatile components such α‐cyperone, nootkatone, ligustilide, senkyunolide A, and senkyunolide I (Liu et al. 2018). In addition, the content of index components in the extracting solution of CX, CR, and 1:2, 1:1, and 2:1 CR‐CX herb pair was measured, respectively. When the CR‐CX herb pair ratio was 1:2, the dissolution rate of ferulic acid was the highest (1.405 mg/g), the dissolution rate of ligustilide was the highest (15.79 mg/g) when the CR‐CX herb pair ratio was 2:1, and the dissolution rate of α‐cyperone was the highest (2.253 mg/g) when the CR‐CX herb pair ratio was 1:1 (Zhang, Wang, et al. 2013; Zhang, Guo, et al. 2013). However, Yang found that the CR‐CX herb pair resulted in a lower content (8.24 mg/mL) of α ‐cyperone compared to CR extraction alone. The discrepancy may be attributed to using water vapor heating for extraction, which may result in partial loss of volatile components, including α ‐cyperone (Yang 2013). Interestingly, the effect of CR‐CX compatibility on α‐cyperone content appears to be method‐dependent. This discrepancy may be explained by differences in extraction methods. Steam distillation, which may cause partial loss of volatile components, whereas solvent extraction coupled with UPLC‐MS/MS. These findings suggested that the choice of extraction method significantly influenced the observed chemical profiles of herb pairs, and that method standardization is essential for meaningful comparisons across studies.

Yang used the rat models of migraine to prove that the absorption and bioavailability of ferulic acid in CX could be promoted significantly (*p* < 0.01) by prolonging the time of retention and action in vivo after compatibility of CR (Yang 2013). Another study also suggested that when the CR‐CX herb pair ratio was 2:1, the ferulic acid pharmacokinetic parameters of *C*_max_, AUC_(0‐*t*)_, AUC_(0‐∞)_, and MRT_(0‐*t*)_ were significantly higher than those of other ratio groups (*p* < 0.05; Zhang 2015) (Table 2).

**TABLE 2:** The main chemical composition of CR‐CX herb pair.

NO.	Name	2D structure	Molecular weight	Molecular formula	CAS
1	Ligustrazine	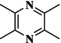	136.190	136.19	1124‐11‐4
2	Ferulic acid	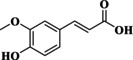	194.184	C_10_H_10_O_4_	1135‐24‐6
3	Ligustilide	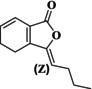	190.238	190.238	81944‐09‐4
4	α‐cyperone	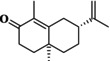	218.335	218.335	473‐08‐5
5	Nootkatone	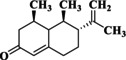	218.335	C_15_H_22_O	4674‐50‐4

#### 3.1.2 Compatibility Effects of the CR‐CX Herb Pair

##### 3.1.2.1 Anti‐Neurogenic Inflammation

**FIGURE 5:**
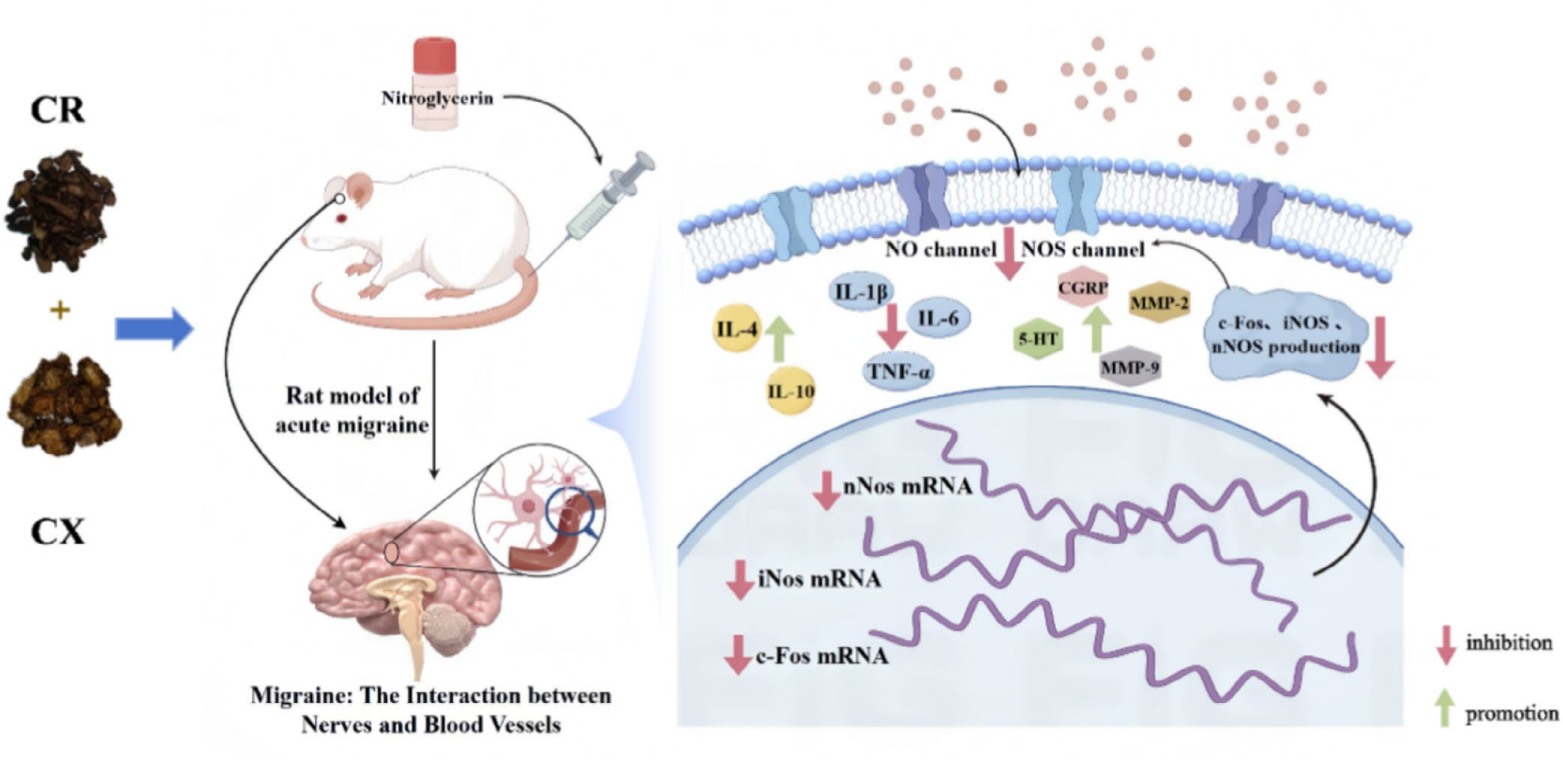
The potential mechanism of anti‐neurogenic inflammation for CR‐CX herb pair.

Neurogenic inflammation is the core of the pathological mechanism of migraine. In a migraine rat model (Kilinc et al. 2020), Wu et al. found that the CR‐CX herb pair could restore abnormal vasoactive substances, including 5‐hydroxytryptamine (5‐HT), Calcitonin gene‐related peptide (CGRP), and Matrix metallopeptidase (MMP‐9) to normal levels, to anti‐neurogenic inflammation. CR‐CX herb pair could also significantly reduce the mRNA and protein expression levels of Fos proto‐oncogene (c‐Fos), Inducible nitric oxide synthase (iNOS), and Neuronal nitric oxide synthase (nNOS) (*p* < 0.05), while CR and CX only inhibited c‐Fos, iNOS mRNA, and protein expression, respectively (Wu et al. 2024). Niu found that the expression of IL‐1β (Interleukin‐1 beta), TNF‐α in the midbrain and plasma of migraine rats was significantly increased (*p* < 0.05), while the expression of 5‐HT was significantly decreased (*p* < 0.05). However, the CR‐CX herb pair could reverse these indicators. Moreover, the CR‐CX herb pair could significantly increase the level of β‐Endorphin (β‐EP) (*p* < 0.05; Niu et al. 2021), which has been proven as the major transmitter of analgesia in the organism (Rhodin et al. 2013), and its decrease would lead to cerebrovascular dysfunction and prolonged headache duration (Misra et al. 2017). Wu et al. used a migraine model with nitroglycerin (Wu et al. 2024). Nitroglycerin can make trigeminal neurons sensitive and increase the inflammatory response (Demartini et al. 2017). Experiments have verified that the CR‐CX herb pair reduced the levels of pro‐inflammatory cytokines, such as interleukin‐1β (IL‐1β) (from 18.97 ng/L to 5.88 ng/L, *p* < 0.0001), IL‐6 (from 133.60 ng/L to 98.27 ng/L, *p* < 0.001), and tumor necrosis factor‐α (TNF‐α) (from 44.86 ng/L to 35.87 ng/L, *p* < 0.001), and increased the amount of anti‐inflammatory cytokines, including IL‐4 (from 20.48 ng/L to 31.11 ng/L, *p* < 0.05) and IL‐10 (from 70.37 ng/L to 96.17 ng/L, *p* < 0.01; Figure 5).

##### 3.1.2.2 Anti‐Depressant Effect

Wei et al. developed a mouse model of depression and conducted a study to examine the effects of alcoholic extracts from the entire YJP formula and individual medicinal herbs, including CR‐CX herb pair, Gardeniae Fructus, Atractylodis Rhizoma, and Massa medicata fermentata. The researchers observed that all of the extracts, except Massa medicata fermentata, reduced the duration of immobility in the hanging tail and forced swimming tests (*p* < 0.05). Furthermore, the entire YJP had the most pronounced effect in enhancing the antidepressant response in mice (Wei et al. 2009). Xue et al. demonstrated that YJP enhances brain‐derived neurotrophic factors (BDNF) expression in the hippocampus through a post‐transcriptional regulatory mechanism (*p* < 0.05) (Xue et al. 2013). Ren showed that the petroleum ether extract of YJP can rapidly alleviate depressive symptoms by enhancing the expression of BDNF and tropomyosin‐related kinase B (TrkB). In a randomized double‐blind controlled trial, Soochim‐tang‐gamibang (SOCG), a Korean formula comprising five traditional Chinese medicines, including CR, is a widely utilized treatment for depression (Ren et al. 2015). The Microbiome‐Gut‐Brain Axis (MGBA) represents a crucial site for bidirectional information exchange between the brain and the intestine (Wang and Kasper 2014). A substantial body of evidence has emerged indicating that the structure of intestinal flora in depressed animals is markedly altered compared to that observed in normal animals (Park et al. 2013). Yu et al. experimentally demonstrated that CHSGS, consisting of CR‐CX herb pair, had a positive regulatory effect on discriminated metabolites in depression model rats, and its regulatory mechanism may be partly through the modulation of intestinal flora, thus altering intestinal flora‐associated fecal metabolites and thus exerting antidepressant effects (Yu et al. 2020).

##### 3.1.2.3 Other Pharmacological Effects

Zhang found that a 2:1 ratio of CR‐CX herb pair could significantly reduce the number of torsions and inhibit uterine contractions, thus improving the pain of primary dysmenorrhea in mice (*p* < 0.01) (Zhang, Wang, et al. 2013; Zhang, Guo, et al. 2013). Yong et al. discovered that the CR‐CX herb pair could diminish the viscosity of whole blood, markedly reduce the electrophoresis time of erythrocytes, and enhance the electrophoresis rate and dispersion of erythrocytes through erythrocyte electrophoresis (Yong and Feng 2000). Zhou et al. found that the Kaixin capsule containing the CR‐CX herb pair was capable of inhibiting collagen synthesis and enhancing myocardial compliance by using a post‐infarction ventricular remodeling model in rats ligating the main trunk of the left coronary artery (*p* < 0.05; Zhou et al. 2002). In summary, the specific parameters and indicators of the changes in pharmacological activity resulting from the combination of CR and CX are compiled and presented in Table 3.

**TABLE 3:** The pharmacotherapeutic properties of CR‐CX.

Extract/Compound	Testing subjects	Types	Dosage	Duration	Major mechanism	Effects	References
CR‐CX = 2:1	Sprague–Dawley rats with nitroglycerin‐induced acute migraine	In vivo	4.375 g/kg/d, i.g.	5 days	c‐fos, iNos, nNos mRNA, c‐Fos, iNOS, nNOS↓, NO↓; IL‐1β, IL‐6, TNF‐α↓; IL‐4, IL‐10↑; 5‐HT↑; CGRP, MMP‐2, MMP‐9↓	Simultaneously regulating neurogenic inflammation and vasoactive substances; Inhibit the NOS/NO pathway	Wu et al. (2024)
Alcoholic extract of CR‐CX	Male Wistar rats with nitroglycerin‐induced migraine	In vivo	6.0, 3.0, 1.5 g/kg bw/day, i.g.	7 days pre‐treatment	5‐HT, β‐EP↑; CGRP, IL‐1β, TNF‐α↓; NO, NOS↓; ET↑	Modulates neurogenic inflammation; Regulates neurotransmitters vasomotor factors	Niu et al. (2021)
Yueju Pill Ethanol Extract	Kunming mice (TST & FST models)	In vivo	30 g/kg/d, i.g.	7 days	—	Significantly reduced tail suspension immobility time (TST) and forced swimming immobility time (FST) in mice. There is no significant impact on independent activity and weight	Wei et al. (2009)
Yueju Ethanol Extrac	Male ICR mice (Learned Helplessness & Tail Suspension Test models)	In vivo	13.5 g/kg, i.g.	Single administration, efficacy evaluation until 24–48 h after administration	BDNF ↑	After 30 min of administration, the expression of BDNF and precursor BDNF proteins in the hippocampus significantly increased, while eEF2 phosphorylation significantly decreased; After 24 h of administration, BDNF protein expression decreased to levels lower than the control group, and eEF2 phosphorylation significantly increased	Xue et al. (2013)
Petroleum ether fraction of Yueju Pill	Kunming mice, molecular analysis	In vivo	6.75 g/kg (low dose), single administration, i.g.	30 min and 24 h post‐administrationf	BDNF, TrkB↑	It related to enhanced neurotrophic signaling and plasticity	Ren et al. (2015)

*Note:* “↑” indicates that it was promotion. “↓” indicates that it was inhibition. “—” indicates that it was not mentioned in the original reference.

#### 3.1.3 Clinical Applications of the CR‐CX Herb Pair

The combination of CR and CX has a good therapeutic effect in the clinic. The majority of the CR‐CX herb pair is incorporated into a compound formula, such as YJP, CHSGP, and so on. Zhang et al. experimentally demonstrated that YJP could improve the BDNF level of depressed patients and, thus, antidepressants, and demonstrated broad therapeutic efficacy in the treatment of depressive disorders (Zhang et al. 2018). Moreover, the disorganization of the intestinal flora of clinically depressed patients has been identified through differentiation tests of patients with clinical depression (Maes et al. 2012). He et al. found that the Zhuifeng Tougu pill, which also contained CR‐CX herb pair, was employed in the treatment of osteoarthritis in patients with chronic kidney disease. The findings indicated that the Zhuifeng Tougu pill was effective in alleviating symptoms such as reduced joint pain and stiffness and demonstrated a lower degree of toxicity to the kidneys (He et al. 2009). Huang et al. demonstrated a significant decrease in *Enterococcus* and *Escherichia coli* and a significant increase in bifidobacteria in patients with depression following the administration of CHSGP. The therapeutic efficacy rate reached 93.3%, providing evidence that CHSGP may exert antidepressant effects through MGBA. Table S1 lists the relevant clinical applications (Huang and Qiu 2017). The inclusion criteria for the data included: (i) confirmation from the doctor that the patient had the appropriate disease. (ii) CR and CX were included in the treatments. (iii) A clear case study was available. Exclusion criteria for the data included: (i) lack of CR and CX doses in the treatment regimen. (ii) Data or other types of research beyond the scope of this work.

CR‐CX herb pair was more common at 1:2 or 2:1 ratios for usage. When the ratio of CR‐CX herb pair was 2:1, the dissolution rate of α‐cyperone and nocardone was high. When the ratio of CR‐CX herb pair was 1:2, the dissolution rate of ferulic acid was high. The CR‐CX herb pair ratio was 3:2 or 1:1, which is suitable for treating digestive system diseases. In the ratio of CR‐CX herb pair of 5:4, it is suitable for improving coronary heart disease. The ratio of CR‐CX herb pair of 1:1 is mostly used for treating liver diseases, such as hepatitis. Although existing studies have systematically clarified the differences in dissolution amounts of CR‐CX under various ratios and identified the optimal ratio for maximum dissolution efficiency, the underlying mechanism remains unelucidated. Such discrepancies may be related to both research methodologies and the inherent properties of the herbal materials: firstly, differences in research methods and instruments across studies—such as variations in HPLC systems, chromatographic columns, extraction solvents, and extraction durations—directly affect the measurement of dissolution amounts; secondly, the specific characteristics of the herbal materials themselves—not only involving multi‐origin issues but also variations in processing methods—can lead to changes in chemical composition and dissolution behavior. Therefore, future research should establish a standardized experimental system. By fixing the herbal species, processing techniques, and research methods, studies can systematically investigate the effects of different compatibility ratios on dissolution behavior, thereby uncovering the chemical interaction mechanisms behind them and providing a scientific basis for optimizing herbal pairing and enhancing quality control. At present, the clinical research on CR‐CX herb pair is primarily focused on its compound prescription, for instance, YJP and CHSGP. However, several issues in the current study merit further examination. In the analysis of the volatile constituent spectrum of CR‐CX herb pair by Wang Huibing et al., there were no newly added components compared to a single traditional Chinese medicine, nor were there any significantly disappeared components in the herb pairs. This indicates a need for continued research on the composition of the two drugs before and after compounding (Wang, Feng, et al. 2017). Furthermore, there is a paucity of studies examining the efficacy of individual components of the CR‐CX herb pair in the treatment of depression, gynecological disorders, and other diseases. The specific mechanisms of action of CR‐CX herb pair in these therapeutic areas remain unclear and warrant further investigation.

### 3.2 CR‐GLJ Herb Pair

GLJ is the dried rhizome of *Alpinia officinarum* Hance. The property of GLJ is acrid and hot (Figure 2B). The main active ingredients of GLJ are volatile oils, flavonoids, sterols, diarylheptanoids, etc. It can warm the stomach, disperse cold, stop the pain, and direct rebellious qi downward. The substance has been demonstrated to possess significant pharmacological properties, including but not limited to: potent anti‐ulcer activity, antibacterial action, antioxidant properties, and the capacity to induce hypoglycemia (Li et al. 2014). It can be used for the treatment of abdominal pain, vomiting, hiccoughs, or diarrhea (Dan et al. 2004). GLJ is primarily cultivated in Xuwen County, Guangdong Province (Yang et al. 2011). In recent years, its cultivation has expanded to Hainan Province through intercropping (alternate planting with forest trees) or understory planting beneath artificial forests. This cultivation approach facilitates its large‐scale cultivation. Furthermore, as a medicinal herb with dual‐use as medicine and food (Yaoshi Tongyuan), GLJ serves not only as a pharmaceutical raw material, but also as an important raw material for condiments and health foods (Pan et al. 2016). It is also widely utilized in cosmetics processing and as a preservative for fruits and vegetables, among other applications (Yan and Lin 2003; Sun et al. 2012). A plethora of compound formulas exist for the CR‐GLJ herb pair, with the most renowned being the Liang Fu Pill (LFP). This pill is primarily employed in the treatment of digestive maladies, including gastric ulcers and chronic gastritis.

#### 3.2.1 Bioactive Components Variation of the CR‐GLJ Herb Pair

CR‐GLJ herb pair was not found to produce new compounds after the extraction of the composition by the method of steam distillation, but the relative concentrations of some compounds were higher, for example, 1,8‐cineol, α‐terpineol, γ‐cadinene, cyperene, cyperenone, and α‐cyperone. However, the relative concentrations of some compounds were reduced or even disappeared, for example, α‐farnesene, cadina‐1(10),4‐diene, β‐eudesmene, isoaromadendrene epox‐ide, and β‐vatirenene in the volatile oil components. The investigation revealed that the oxygenated monoterpenes present in the volatile oils of CR‐GLJ herb pair was predominantly derived from GLJ, while the oxygenated sesquiterpenes were primarily derived from CR. These compounds were found to possess significant biological activities (Qu et al. 2021). The total flavonoid content of the pairs was determined spectrophotometrically at different ratios (CR: GLJ = 9:1, 5:1, 7:1, 3:1, 1:1, 1:3, 1:7, 1:5, 1:9), and it was found that the highest total flavonoid content was found in the ratio of 3:1 for CR: GLJ.

After comparing CR‐GLJ herb pair in the pharmacokinetic study of rat plasma using high‐performance liquid chromatography and gas chromatography, DAS2.0 analysis was used, combined with the fitting analysis of the atrial model. It was found that after pairing with CR, the peak time of the galangal extract was advanced. The amount of absorption was increased, which proved that CR could promote the absorption of galangal, reflecting the rationality of the pairing (Wang et al. 2009; Table 4).

**TABLE 4:** The main chemical composition of CR‐GLJ herb pair.

NO.	Name	2D structure	Molecular weight	Molecular formula	CAS
1	1,8‐cineol	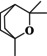	154.249	C_10_H_18_O	470‐82‐6
2	α‐terpineol	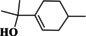	154.249	C_10_H_18_O	24302‐23‐6
3	γ‐cadinene	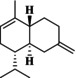	204.351	C_15_H_24_	5957‐55‐1
4	Cyperene	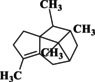	204.351	C_15_H_24_	2387‐78‐2
5	Cyperenone	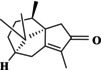	218.335	C_15_H_22_O	3466‐15‐7
6	α‐cyperone	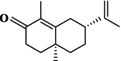	218.335	C_15_H_22_O	473‐08‐5
7	cadina‐1 (10), 4‐diene	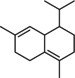	204.351	C_15_H_24_	16729‐01‐4

#### 3.2.2 Compatibility Effects of the CR‐GLJ Herb Pair

##### 3.2.2.1 Anti‐Gastric Mucosal Injury

Gastric ulcers, as a common manifestation of the inflammatory response, can exacerbate gastric mucosal damage through the release of the pro‐inflammatory cell mediator IL‐6 by macrophages in the ulcerated area. CR‐GLJ herb pair significantly reduced the levels of IL‐6, TNF‐α, and Cyclooxygenase‐2 (COX‐2) as verified by in vitro experiments (*p* < 0.05). In addition, CR‐GLJ herb pair has a protective effect on the gastric mucosa, inhibiting the protein expression of Nuclear Factor kappa Bp65 (NF‐κBp65), COX‐2, and Transient Receptor Potential Vanilloid 1 (TRPV1) and decreasing the concentration of IL‐6 and TNF‐α, thus reducing pain. Animal studies have shown that CR‐GLJ herb pair could protect mice from ethanol‐induced gastric mucosal damage by inhibiting inflammation and analgesia (Qu et al. 2021). After establishing a mouse model of gastric ulcer induced by anhydrous ethanol, it was found that compared with CR or GLJ alone, CR‐GLJ herb pair significantly reduced the ulcer index (*p* < 0.01) and significantly reduced the levels of IL‐6 and IL‐1β (*p* < 0.01; Chai and Li 2023).

**FIGURE 6:**
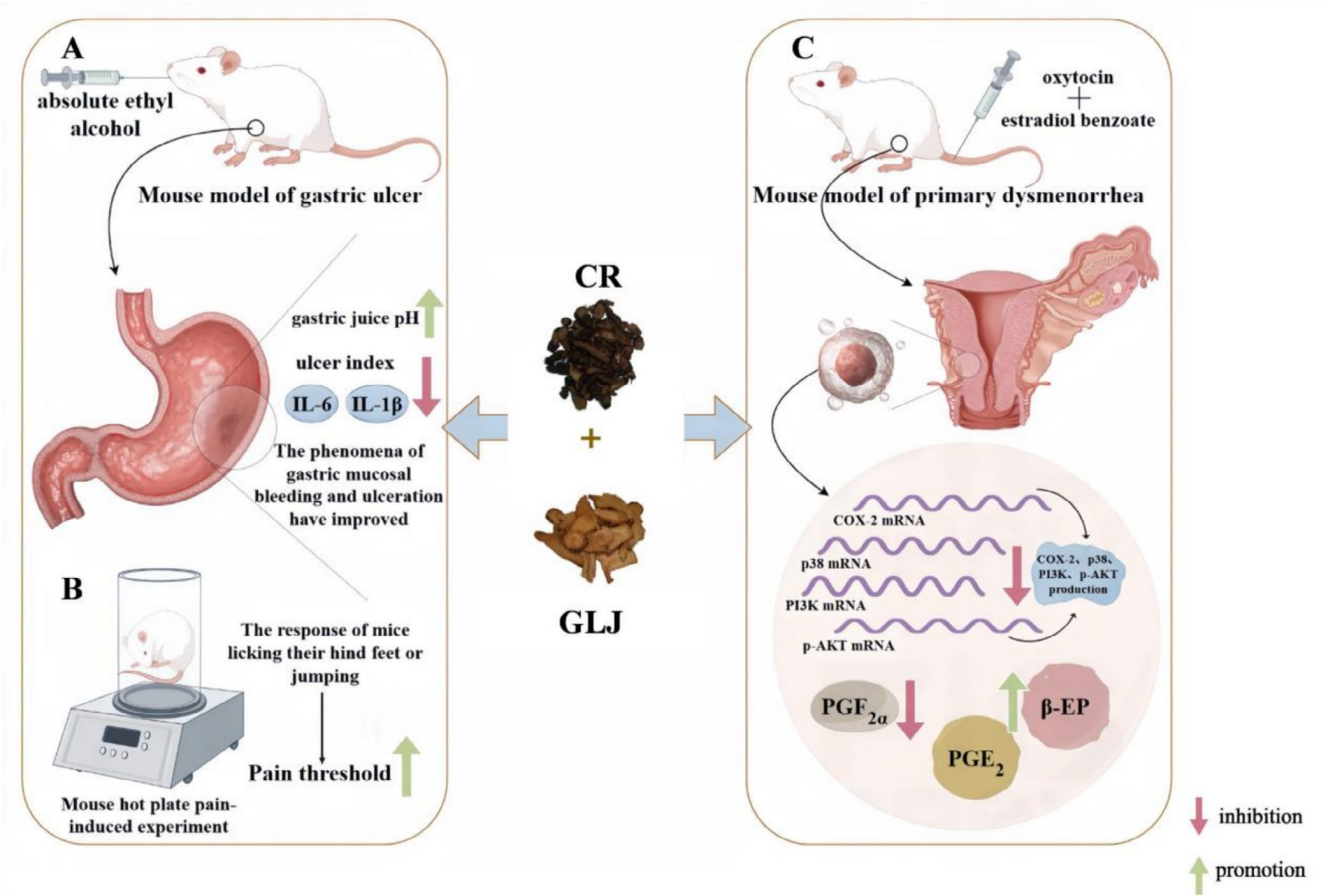
The potential mechanism of CR‐GLJ herb pair. (A) Anti‐gastric ulcer mechanism, (B) Mechanisms of analgesia, (C) Anti‐PD mechanisms.

Wei Yan Ning Pill (WYNP) containing CR‐GLJ herb pair, which is a commonly used proprietary Chinese medicine in clinical practice, could resist gastric mucosal damage. Among it, the ratio of CR: GLJ is 2:1. Dai et al. modeled the gastric mucosal damage caused by anhydrous ethanol in rats and verified that the mechanism of action of WYNP in ameliorating the gastric mucosal damage was related to the ability of the drug to increase the expression of the tight junction protein Claudin‐7, the adhesion junction proteins E‐cadherin and β‐catenin, the mucin proteins Mucin 5AC (MUC5A) and Mucin 1 (MUC1), and the transcription factor sex‐determining region Y‐box2 (SOX2) (*p* < 0.05) (Dai et al. 2024). MUC1 and MUC5AC, as mucins secreted on the mucosal surface, are key defense factors in the gastric mucosal defense system. SOX2 regulates mucin secretion and gastric epithelial cell differentiation and plays an important role in the maintenance of the normal phenotype and function of mucus cells (Li et al. 2004; Thompson et al. 2018) (Figure 6A).

##### 3.2.2.2 Analgesic Effect

Chai et al. established a hot‐plate analgesic test and found that CR‐GLJ herb pair significantly elevated the pain threshold and had a favorable analgesic effect compared with CR or GLJ alone (*p* < 0.01; Chai and Li 2023). A primary dysmenorrhea (PD) model in mice was prepared by combining estradiol benzoate and oxytocin. It was verified that CR‐GLJ herb pair could significantly reduce the number of writhing in mice (*p* < 0.01), alleviate the uterine tissue edema and inflammation in mice with PD, reduce the level of prostaglandin F_2α_ (PGF_2α_) (from 171.00 ± 19.40 pg·mL^−1^ to 101.64 ± 23.60 pg·mL^−1^) in the serum, elevate the level of prostaglandin E_2_ (PGE_2_) (from 186.81 ± 31.17 pg·mL^−1^ to 320.51 ± 44.14 pg·mL^−1^) and β‐EP (from 290.02 ± 16.69 pg·mL^−1^ to 377.82 ± 55.98 pg·mL^−1^), and inhibit the protein expression of COX‐2 (from 2.40 ± 0.68 pg·mL^−1^ to 0.88 ± 0.14 pg·mL^−1^), p38 (from 1.17 ± 0.08 pg·mL^−1^ to 0.65 ± 0.15 pg·mL^−1^), PI3K (from 1.52 ± 0.17 pg·mL^−1^ to 0.51 ± 0.08 pg·mL^−1^), and p‐AKT (from 1.45 ± 0.08 pg·mL^−1^ to 0.63 ± 0.14 pg·mL^−1^). The mechanism of pain relief in PD may be attributed to the role of PI3K/AKT、MAPK、PTGS2 and other pathways (Huang et al. 2023; Figure 6B,C). In summary, the specific parameters and indicators of the changes in pharmacological activity resulting from the combination of CR and GLJ are compiled and presented in Table 5.

**TABLE 5:** The pharmacotherapeutic properties of CR‐GLJ.

Extract/Compound	Testing subjects	Types	Dosage	Duration	Major mechanism	Effects	References
Essential Oil of CR‐GLJ	BALB/c mice (ethanol‐induced gastric ulcer)	In vivo	780 (Low) & 1560 mg/kg (High), p.o.	7 days	NF‐κBp65, COX‐2, TRPV1, IL‐6, TNF‐α↓	Significantly reduced gastric mucosal injury (ulcer index), inhibited inflammatory response, and provided analgesia (increased pain threshold in hot plate test)	Qu et al. (2021)
Aqueous extract of CR‐GLJ	KM mice with ethanol‐induced gastric ulcer	In vivo	0.8 g·kg^−1^, i. g.	7 days	IL‐6↓, IL‐1β↓	Significantly protected against ethanol‐induced gastric ulcers. The effect was stronger than either herb used alone	Chai and Li (2023)
Weiyangning Pill	SD rats with ethanol‐induced gastric mucosal injury	In vivo	3 & 1.5 g·kg^−1^, i. g.	Single pre‐treatment (2 h before ethanol)	Claudin‐7↑, E‐cadherin↑, β‐catenin↑, MUC1↑, MUC5AC↑, SOX2↑	Significantly reduced gross and pathological injury scores of gastric mucosa. Protected gastric mucosal barrier integrity	Dai et al. (2024)
Ethanol extract of CR‐GLJ	ICR female mice with estradiol benzoate & oxytocin‐induced PD	In vivo	0.9, 0.3, 0.1 g·kg^−1^, i.g	7 days	PGF2α↓, PGE2↑, β‐EP↑. COX‐2 protein/mRNA↓, p38 protein/MAPK14 mRNA↓, PI3K/p‐PI3K protein/mRNA↓, p‐AKT protein↓	Significantly reducing the number of writhing episodes and increasing the latency period in PD mice, it also demonstrated analgesic and anti‐inflammatory effects on primary dysmenorrhea	Huang et al. (2023)

*Note:* “↑” indicates that it was promotion. “↓” indicates that it was inhibition “—” indicates that it was not mentioned in the original reference.

#### 3.2.3 Clinical Applications of CR‐GLJ Herb Pair

CR‐GLJ herb pair is primarily employed in the treatment of analgesic and digestive disorders, encompassing conditions such as gastric ulcers and PD. The classic clinical formula for LFP has been developed into a modern Chinese patent medicine, WYNP, which can be used in the treatment of gastric ulcers and other digestive disorders, and can be used in the treatment of gastric ulcers and other digestive disorders. Furthermore, as a specialized therapeutic modality within the domain of TCM, the combination of external TCM treatment has demonstrated notable clinical efficacy. For instance, Qu et al. conducted a study that observed the efficacy of auricular acupressure in conjunction with traditional Chinese medicine hot compresses for 46 cases of stomach and epigastric pain. The treatment regimen incorporated auricular acupressure by Vaccariae semen and CR‐GLJ herb pair, among others, utilizing heated and pasted crude salt on specific acupoints. The total therapeutic efficacy rate achieved was as high as 95.65% (Qu et al. 2021).

CR‐GLJ herb pair is mostly used in the treatment of gastrointestinal discomforts, the total flavonoids and volatile oils extracted in the ratio of 3:1 is the highest, and in the pharmacodynamic experiments of the pharmacological effects of the optimal, can significantly inhibit ulcers (*p* < 0.01), with an inhibition rate of 74.67%, and slow down the gastrointestinal propulsion, so the clinic also uses this ratio. Moreover, CR‐GLJ herb pair can be used in functional foods for gastric mucosa protection—the Chinese patent application “Health food composition with auxiliary protective function for gastric mucosa and its preparation method” (Application date: February 6, 2013; Published application number: CN102907673A) utilizes a formulation containing herbs such as Salviae Miltiorrhizae Radix et Rhizoma (Danshen) and Bletillae Rhizoma (Baiji). It can also be supplemented with external treatments such as auricular acupoint pressure and hot compresses, which provide a wide variety of therapies and better clinical results (Wang et al. 2009). Furthermore, we have compiled Table S2 of some clinical application cases of CR‐GLJ, as detailed in the Supporting Information. The inclusion criteria for the data included: (i) confirmation from the doctor that the patient had the appropriate disease. (ii) CR and GLJ were included in the treatments. Exclusion criteria for the data included: (i) lack of CR and GLJ doses in the treatment regimen. (ii) Data or other types of research beyond the scope of this work.

### 3.3 CR‐AY Herb Pair

AY is the dried leaves of *Artemisia argyi* Levl. et Vant. in the family Asteraceae (Figure 2C). Artemisiae Argyi Folium main chemical constituents include terpenes, flavonoids, phenylpropanoids, steroids, trace elements, etc. (Lan et al. 2020; Li et al. 2016). Modern pharmacological effects include analgesic, antibacterial and antiviral, anti‐inflammatory, anti‐tumor, etc. (Li et al. 2016). Its traditional medicinal values are that it warms the womb and stops bleeding, disperses cold and alleviates pain, eliminates dampness, and stops itching. Its vinegar stir‐frying charcoal can also enhance the effect of hemostasis (Dan et al. 2004). Currently in China, the cultivation of AY and its product processing are widely distributed, primarily concentrated in two major industrial clusters: Qichun County, Hubei Province and Nanyang City, Henan Province (Ke and Zhang 2018; Li, Liu, and Li 2018). Additionally, in agriculture, AY can be utilized: As a feed additive in livestock farming. Research by Xia et al. has demonstrated that adding AY to the total mixed ration (TMR) of dairy cows can increase milk yield and enhance milk flavor. As a botanical pesticide for crop pest and disease control (Ma et al. 2024). It exhibits characteristics of being environmentally friendly, innovative, and highly effective, contributing to sustainable agricultural development. This application holds significant potential for future expansion. Furthermore, AY is edible with a long history of culinary use (Wang et al. 2024). While preserving its traditional culinary practices, modern applications have developed novel health‐preserving functional foods, such as mugwort leaf tea and mugwort‐infused pastries. For example, the Chinese patent application “A chocolate‐based herbal compound formulation for the treatment and alleviation of female menstrual cycles and menstrual syndrome” (Application date: January 27, 2016; Published application number: CN105267906A) utilizes a formulation containing herbs such as Angelicae Sinensis Radix (Danggui), Cyathulae Radix (Niuxi). Notably, this formulation also incorporates the CR‐AY herb pair. Furthermore, CP, a medicinal and edible herb, is included, which is frequently used in combination with CR in clinical practice. For CR‐AY herb pair, there are well‐established formulas, including the Ai Fu Nuan Gong Pill (AFNGP) and the Zhu Fu Zi Pill (ZFZW), primarily employed in the treatment of gynecological ailments.

#### 3.3.1 Bioactive Components Variation of the CR‐AY Herb Pair

It has been determined that CR and AY contain substantial quantities of volatile oils. Experimentally, the components of the volatile oil of the CR‐AY herb pair were mainly derived from AY, and some components of the pairs disappeared, such as 1,6‐dimethylhepta‐1,3,5‐triene, and some new components were generated, such as 4‐methyl‐1‐(1‐methylethyl)‐bicyclo[3.1.0] hexan‐3‐one. In the clinical use of TCM, both CR and AY have the processing form of vinegar products, and the composition of the two herb pairs has also changed significantly after vinegar processing (He et al. 2015). Changes in volatile oil composition of the CR‐AY herb pair before and after vinegar was processed were identified by GC–MS based on a combination of AMDIS and retention index by Fan et al. It was found that the content of most of the low‐boiling compounds decreased in the vinegared CR‐AY herb pair (mainly attributed to AY), while there was no significant change in the high‐boiling compounds (mainly attributed to CR), but the content of α‐cyperone increased. Secondly, the compounds in the volatile oils of the pairs that increased significantly after the vinegar preparation were mostly deoxygenated sesquiterpenes, long‐chain fatty alcohols, and long‐chain fatty ketones, while the content of terpene oxidized derivatives decreased. The specific mechanism may be that the hydrogen bonds of the relatively more polar low‐boiling point terpene oxidized derivatives are more easily dispersed in acetic acid solution, more readily exuded in plant tissues, and ultimately volatilized when stir‐fried in vinegar. The deoxy sesquiterpenoids that cannot form hydrogen bonds and the long‐chain fatty alcohols and long‐chain fatty ketones whose polar groups are easily encapsulated by long chains are more likely to remain in the plant tissues, and the volatilization rate is slower than that of the former, so the content is higher (Fan et al. 2017). Naturally, it is also possible that there may be compositional transformations that increase or decrease the content, requiring further experimental studies.

#### 3.3.2 Compatibility Effects of the CR‐AY Herb Pair

##### 3.3.2.1 Analgesic Effect

Mao et al. conducted electrical stimulation and acetic acid analgesic experiments in groups of mice and found that CR‐AY herb pair and individual herbs had significant analgesic effects on mice (*p* < 0.01; Mao et al. 1998).

##### 3.3.2.2 The Impact on the Blood

Mice after CR, AY gavage, it was determined that the CR‐AY herb pair could elicit a substantial increase in platelet count (*p* < 0.01) while exhibiting no significant impact on heme levels. CR‐AY herb pair also shortens clotting time, which may be related to an increase in platelet counts (Mao et al. 1998).

##### 3.3.2.3 Clinical Applications of CR‐AY Herb Pair

Clinical studies have shown that the CR‐AY herb pair can treat primary infertility, dysmenorrhoea, premature ovarian failure, and other gynecological disorders. The total effective rate of AFNGP with the CR‐AY herb pair as the main herb in the treatment of 33 patients with primary infertility was 100% (Chen and Li 1989). Adopting the artificial menstrual cycle method of traditional Chinese medicine, using the CR‐AY herb pair and other traditional Chinese medicines to treat premature ovarian failure for 6 months, while the control group was treated with Liu Wei Di Huang Pill (LWDHP), the results showed that the total effective rate of the traditional Chinese medicine group was 80% in 60 cases, which was better than that of the control group (*p* < 0.05), and there were no adverse reactions (Hui and Sun 2015). LWDHP is mainly used to nourish kidney yin and can be used to treat women with irregular menstruation (Gong 2018), premature ovarian failure, and other diseases (Table S3). The inclusion criteria for the data included: (i) confirmation from the doctor that the patient had the appropriate disease. (ii) CR and AY were included in the treatments. Exclusion criteria for the data included: (i) lack of CR and AY doses in the treatment regimen. (ii) Data or other types of research beyond the scope of this work.

In clinical practice, CR‐AY is commonly used in a 2:1 ratio to treat dysmenorrhea, which may be related to the secretion of β‐EP (Wang et al. 2003). 2:1 ratio can also be applied to functional dysfunctional uterine bleeding to increase platelet count (Mao et al. 1998). Furthermore, the vinegar processing method could change the chemical composition and enhance the efficacy of CR‐AY herb pair. However, the optimal processing parameters (e.g., vinegar concentration, soaking duration) and their mechanistic impact on pharmacokinetics require further standardization and validation.

### 3.4 CR‐CP Herb Pair

CP is the dried mature pericarp of the orange *Citrus reticulata* Blanco and its cultivated varieties (Figure 2D). CP contains mainly volatile oils, flavonoids, alkaloids, and other substances. It could regulate the qi, adjust the middle, and relieve the diaphragm. Its main pharmacological effects are anti‐inflammatory and antibacterial, antioxidant, anti‐tumor, anti‐thrombotic, and vascular protection *etc*. (Xu et al. 2022). Additionally, CP can be used as an ingredient in health foods, primarily to regulate spleen and stomach function and promote digestion (Li, Li, et al. 2025; Cao and Pan 2022). CR‐CP herb pair could enhance qi‐promoting effects. Jia Wei Ping Wei San (JWPWS) and Xiang Su San (XSS) are its classic remedies in clinical practice.

#### 3.4.1 Bioactive Components Variation of the CR‐CP Herb Pair

Li et al. extracted CR‐CP herb pair volatile oil and used GC–MS technique to detect the main chemical components of the volatile oil of the herb pairs, mainly 1,2,3,4,5,6‐hexahydro‐1,1,5,5‐tetramethyl‐7H‐2,4α‐methyl‐7‐ketone, (4aR,5S)‐4α‐2,5‐dimethyl 3‐propan‐2‐ylidenemethyl‐5,6,7,8‐tetrahydro‐4H‐naphthalen‐2‐one and eudesmol‐2‐ketone and eucalyptol. However, 1,2,3,4,5,6‐hexahydro‐1,1,5,5‐tetramethyl‐7H‐2,4α‐methyl‐7‐one and (4aR,5S)‐4α‐2,5‐dimethyl‐3‐propan‐2‐ylidenemethyl‐5,6,7,8‐tetrahydro‐4H‐naphthalen‐2‐one have not been reported in either CP or CR volatile oils and are presumed to be new to the pair (Li, Lu, et al. 2018). It may be produced by certain chemical reactions, such as reduction, hydrolysis, and oxidation, or physical changes, such as solubilizing effects, that occur when the two are combined in a decoction of both (She et al. 2011).

#### 3.4.2 Clinical Applications of CR‐CP Herb Pair

The clinical utilization of the Chen Pi Xiang Fu Yin (CPXFY) has yielded noteworthy outcomes in the treatment of common gynecological diseases such as mammary abscess. A comprehensive analysis encompassing 36 cases reveals an impressive success rate of 94%, with the majority of cases demonstrating resolution within a span of 5 days following the administration of the drink (Zeng and Yang 1991). CR‐CP herb pair can also be used in the treatment of stomach and epigastric pain, such as in the case of a 45‐year‐old woman, stomach distension, often hidden pain recurrent, ultrasound reality of its chronic cholecystitis, the use of the herb pairs, and with the symptoms of additional drugs such as CH, Aurantii Fructus (Zhiqiao), Citri Sarcodactylis Fructus (Foshou), cured after half a month of treatment (Li 2004) (Table S4). The inclusion criteria for the data included: (i) confirmation from the doctor that the patient had the appropriate disease. (ii) CR and CP were included in the treatments. (iii) A clear case study was available. Exclusion criteria for the data included: (i) lack of CR and CP doses in the treatment regimen. (ii) Data or other types of research beyond the scope of this work.

CR‐CP herb pair is primarily utilized in the treatment of gynecological and digestive system diseases. In clinical practice, CR‐CP is often used in a 3:2 ratio to increase the levels of plasma motilin and acetylcholinesterase, and is used to treat functional dyspepsia (Xue et al. 2020). And treat antral gastritis inflammation in a 1:1 ratio (Chen 2009). Addressing these gaps through integrated systems pharmacology approaches and standardized animal models of gastrointestinal dysregulation is essential for developing evidence‐based clinical protocols and facilitating modern drug development from this classical herb pair.

In addition to the four aforementioned CR herb pairs that are commonly used for both medicinal and food purposes, there are also herbs with potential for development as food, such as Atractylodis Rhizoma (Cangzhu in Chinese, CZ) and Bupleuri Radix (Chaihu in Chinese, CH), which are also commonly used as clinical herb pairs. A further summary of these herbs can be found in the Supplementary Materia, which containing Figure S1–S3 and Tables S5–S7.

## 4 Discussion

This review is the first to integrate the traditionally distinct domains of culinary processing and herb‐pair compatibility within the context of functional food development from CR. Our synthesis of the literature suggests that food‐grade processing methods, including the use of vinegar, wine, and salt, may enhance CR's bioactivity through mechanisms such as, flavonoid deglycosylation and modulation of volatile oil constituents. Furthermore, the systematic analysis of herb pairs presented here indicates that specific ratios of CR with food‐compatible botanicals can yield distinct chemical profiles and pharmacological outcomes, offering a valuable reference for evidence‐based nutraceutical formulation. By reframing an invasive agricultural weed as a potential source of high‐value health products, this work highlights a possible strategy for sustainable agricultural byproduct upcycling. Collectively, these observations help bridge traditional herbal knowledge with modern food science.

Current research on processing CR with culinary excipients (e.g., vinegar, wine, and salt) remains predominantly anchored in TCM processing principles. While these methods enhance its bioactivity, greater innovation could emerge from integrating techniques adapted from food fermentation, such as those used in wine production, where controlled microbial fermentation and aging significantly modify bioactive profiles. Unlike standardized TCM practices, culinary‐inspired processing lacks uniform guidelines regarding excipient selection, dosage optimization, or processing parameters, leading to inconsistent outcomes. For instance, while vinegar‐processed CR has demonstrated improved anti‐inflammatory effects, the concentration and type of acetic acid, fermentation duration, and synergistic interactions with other ingredients remain underexplored. Common processing adjuvants, such as vinegar and alcohol, are themselves fermented foods. The quality of the raw materials and the fermentation process directly influence the chemical composition of these adjuvants, such as ethanol content, total acidity, and total esters, which in turn affects the transformation of CR herbal components like α‐cyperone during processing and the final product quality. Therefore, establishing comprehensive food standards across from raw materials to finished products is crucial for ensuring the stability of subsequent processing steps. This entails creating a systematic and comprehensive regulatory framework: one that encompasses not only processing raw materials like CR, but also integrates the production of processing adjuvants into a standardized system. For instance, alcohol used in processing must have clearly defined raw material standards, fermentation process parameters, food safety indicators, and quality specifications for processing. Future studies should establish evidence‐based protocols by systematically comparing variables such as alcoholic fermentation versus acidic marination, while also assessing scalability for industrial applications. This shift toward food‐grade processing standards could bridge the gap between empirical TCM knowledge and reproducible functional food development.

The synergistic effects of CR when co‐administered with other medicinal and culinary herbs, such as GLJ, have been preliminarily demonstrated in pharmacological studies. However, there remains a significant research gap in determining the precise herb‐herb interaction ratios that maximize therapeutic efficacy for specific health benefits. Existing data indicate that certain combinations of CR with compatible botanicals exhibit enhanced biological activities, including hepatoprotection, gastric motility promotion, antidepressant effects, and neurotrophic modulation, all of which represent important potential applications in functional food development. Despite these promising findings, the specific bioactive constituents responsible for the observed synergies, their dose–response relationships, and optimized combination ratios require further elucidation. For example, while the CR‐GLJ herb pair has shown improved anti‐ulcer activity, whether the optimal efficacy occurs at a 1:1, 2:1, or other specific ratio remains to be scientifically validated through systematic studies. Additionally, the underlying molecular mechanisms of these herbal interactions at target sites need thorough investigation.

From an application perspective, the conversion of these herbal combinations into commercial functional food products necessitates a more standardized approach. Developing an agricultural upcycling model for CR would provide significant value, transforming this invasive plant species from a traditional medicinal material into a versatile functional food ingredient. Future research should focus on establishing evidence‐based combination ratios (such as CR‐GLJ at 3:1 for gastric mucosa protection, CR‐CX at 1:1 for liver protection) while concurrently investigating how processing methods influence the bioactive metabolite profiles of these herb pairs. This dual‐focused strategy would effectively bridge traditional herbal knowledge with modern food science, potentially positioning CR as a valuable nutraceutical ingredient suitable for dietary supplements as well as fortified conventional food products. While the traditional efficacy of CR provides scientific justification for its development as a functional food, its safety requires further risk assessment. The transition between therapeutic efficacy and toxicity depends on dosage. CR's key active component, α‐cyperone, exhibits pronounced dose‐dependent effects in cellular experiments: concentrations below 60 μM demonstrate anti‐inflammatory protective effects, whereas 120 μM causes a slight reduction in cell viability (Huang et al. 2018). This study indicates that precisely defining CR's safe range is a primary task in developing CR‐related foods. However, substantial gaps remain regarding CR's potential toxicity and allergenicity. Several researchers explicitly call for “further investigation into subchronic toxicity and drug interactions”, while acknowledging that “not all constituents within the compound formulation have been identified” (Sutalangka and Wattanathorn 2017). As a complex herbal preparation, proteins, polysaccharides, and other constituents within CR readily serve as potential allergens, posing risks to consumers with allergic predispositions. This uncertainty creates regulatory hurdles. Consequently, future research must intensify investigations into CR's potential toxicity and allergenicity while proactively addressing regulatory barriers. This approach will determine whether products can legally enter the market, secure long‐term consumer trust, and ultimately ensure the success of the entire development programme.

Emerging nanoparticle supplementation strategies offer transformative potential for enhancing the bioavailability and targeted delivery of CR‐derived compounds, particularly when combined with culinary processing methods. While traditional vinegar, wine, and other excipient‐based processing modifies CR's chemical profile, nano‐encapsulation technologies could amplify these effects by protecting thermolabile constituents during food manufacturing and improving intestinal absorption. For instance, 4‐aminophenyl β‐D‐galactopyranoside (Gal‐NH2)/mulberry leaf polysaccharides‐lysozyme (Gal‐MPL) nanoparticles loaded with CR's active luteolin via amide reaction, self‐assembly process, and electrostatic interaction have been demonstrated that the nanoparticles could hepatic‐target and enhance action on liver tissue by specific recognition of asialoglycoprotein receptor (Li et al. 2023). This suggested potential synergy with the hepatoprotective herb‐pair compatibility combinations discussed earlier. However, current research remains confined to pharmaceutical applications, neglecting opportunities to adapt these carriers for food‐grade systems. Future studies should evaluate edible nanoparticle formulations, such as chitosan or alginate‐based, co‐encapsulating CR and its synergistic herb pairs, with rigorous assessment of processing stability in fermented or thermally processed functional foods. This approach would concurrently address the standardization challenges in herbal processing while creating clinically translatable nutraceuticals.

Despite the promising findings summarized in this review, several limitations in the current literature warrant critical consideration. The existing literature upon which this review is based is limited in quantity, particularly concerning research into CR culinary processing and the development of associated functional foods, where studies remain insufficient and reports scarce. This has, to some extent, constrained a comprehensive assessment of CR's potential applications in food development. Also, most pharmacological studies have been conducted using crude extracts rather than standardized fractions or pure compounds, making it difficult to attribute observed effects to specific constituents. Existing studies predominantly focus on preliminary investigations into chemical composition and pharmacological mechanisms. Not only is there a lack of large‐scale clinical trial data, but most clinical and animal experiments also exhibit methodological shortcomings, such as the omission of critical details including dosage, extraction solvents, and processing temperatures. Consequently, future research should concentrate on the development of foods incorporating CR and its associated medicinal compounds, advance clinical trials to systematically evaluate safety, emphasize the comprehensive presentation of experimental methodology details, and persistently explore standardized and expanded applications of CR within food processing.

## 5 Conclusion

This review highlights the potential of culinary processing and synergistic herb‐pair strategies to enhance the therapeutic efficacy of CR, positioning it as a sustainable, multi‐functional ingredient. Traditional methods such as vinegar, wine, and salt‐processing modify its chemical profile, increasing bioactive compounds like α‐cyperone and improving bioavailability. Optimized herb‐pair ratios further demonstrate how food‐compatible botanical synergies can amplify pharmacological outcomes. Integration with modern food science, including nanoencapsulation in edible carriers, offers scalable solutions to stability and absorption challenges in functional foods. Future research must focus on standardizing culinary processing parameters, validating herb‐pair mechanisms through multi‐omics approaches, and translating these findings into industrial applications that align with sustainable development goals by repurposing this agricultural pest into high‐value health products. By bridging traditional knowledge with contemporary food technology, this work provides a framework for developing next‐generation nutraceuticals that leverage CR's dual potential as both a food and medicine while addressing global health and environmental challenges.

## Author Contributions

**Yuehan Liu:** writing – original draft, data curation. **Liyuan Xu:** visualization. **Liting Lin:** writing – review and editing. **Tianhui Gao:** writing – review and editing, conceptualization, project administration, supervision. **Wan Liao:** writing – review and editing, project administration, supervision. **Maoyuan Jiang:** writing – review and editing.

## Funding

This work was supported by the National Natural Science Foundation of China (NO. 82505032), “Youth Innovation Team Plan” of Universities in Shandong Province (NO. 2025KJJ024), 2025 Research Project on Traditional Chinese Medicine Monitoring and Statistics (NO. 2025JCTJA16) and 2025 Shandong Province Youth Research Project for College Students (NO. WL‐SQD25063).

## Conflicts of Interest

The authors declare no conflicts of interest.

## Supporting information


**Figure S1:** Schematic diagram of CZ plant.
**Figure S2:** Schematic diagram of CH plant.
**Figure S3:** PPI and H‐C‐T network analysis of 44 potential therapeutic targets for CXP in HCC (Qing et al. 2022).
**Table S1:** Clinical application of CR‐CX herb pair.
**Table S2:** Clinical application of CR‐GLJ herb pair.
**Table S3:** Clinical application of CR‐AY herb pair.
**Table S4:** Clinical application of CR‐CP herb pair.
**Table S5:** The main chemical composition of CR‐CZ herb pair.
**Table S6:** Clinical application of CR‐CZ herb pair.
**Table S7:** Clinical application of CR‐CH herb pair.

## Data Availability

The authors have nothing to report.

## References

[fsn371808-bib-0001] Cao, X. M. , and S. Y. Pan . 2022. “Progress in Research on Secondary Metabolites and Biological Activity of Medicinal and Edible Citrus Plants.” Food Science 43, no. 23: 305–315.

[fsn371808-bib-0002] Chai, X. , and X. L. Li . 2023. “Study on Anti‐Gastric Ulcer and Analgesic Effects of Gaoliangjiang‐Xiangfu.” Acta Chinese Medicine and Pharmacology 51, no. 6: 38–42.

[fsn371808-bib-0003] Chen, H. L. 2009. “Banxia Xiexin Decoction in the Treatment of 102 Cases of Antral Gastritis.” Journal of Practical Traditional Chinese Medicine 25, no. 9: 600–601.

[fsn371808-bib-0004] Chen, J. F. , and D. D. Li . 1989. “Summary of 33 Cases of Primary Infertility Cured by Aifu Nuangong Pill.” Jiangsu Journal of Traditional Chinese Medicine 8: 10–11.

[fsn371808-bib-0006] Chen, Y. , N. Li , D. Wang , et al. 2022. “Analysis of Raw and Processed Cyperi Rhizoma Samples Using Liquid Chromatography‐Tandem Mass Spectrometry in Rats With Primary Dysmenorrhea.” Journal of Visualized Experiments 190: e64691. 10.3791/64691 36622033

[fsn371808-bib-0007] Chen, Y. L. , N. Li , R. Chu , et al. 2023. “Historical Evolution and Research Status of Processing of Cyperi Rhizoma.” Natural Product Research and Development 35, no. 4: 722–731.

[fsn371808-bib-0011] Dai, X. Z. , Z. M. Zhu , X. L. Wang , et al. 2024. “Effect Mechanism of Chniese Patent Medicine Weiyangning Pill on Preventing and Treating Gastric Mucosal Injury in Rats.” Herald of Medicine 43, no. 12: 1904–1912.

[fsn371808-bib-0012] Dan, B. , C. Steven , S. Erich , et al. 2004. Chinese Herbal Medicine: Materia Medica. 3th ed. Eastland Press, Inc.

[fsn371808-bib-0013] Demartini, C. , C. Tassorelli , A. M. Zanaboni , et al. 2017. “The Role of the Transient Receptor Potential Ankyrin Type‐1 (TRPA1) Channel in Migraine Pain: Evaluation in an Animal Model.” Journal of Headache and Pain 18, no. 1: 94. 28884307 10.1186/s10194-017-0804-4 PMC5589714

[fsn371808-bib-0014] Deng, Y. , C. H. Zhang , H. N. Zhang , et al. 2011. “Effects of Chaihu Shugan Powder on the Behavior and Expressions of BDNF and TrkB in the Hippocampus, Amygdala, and the Frontal Lobe in Rat Model of Depression.” Chinese Journal of Integrated Traditional and Western Medicine (Chinese Edition) 31, no. 10: 1373–1378. 22097208

[fsn371808-bib-0015] Fan, K. L. , H. Cai , Y. Duan , et al. 2017. “Qualitative Investigation on Changes of Chemical Constutuents From Volatile Oils Between Crude and Vinegar‐Processed Cyperi‐Rhizoma‐Artemisae Argyi Folium Herb‐Pair Based on AMDIS and Retention Index Coupled With DC‐MS Analysis.” Journal of Nanjing University of Traditional Chinese Medicine 33, no. 3: 301–307.

[fsn371808-bib-0016] Gong, Z. Y. 2018. “Clinical Research of Modified Liuwei Dihuang Pills in the Treatment of 50 Cases Irregular Menstruation.” Clinical Journal of Traditional Chinese Medicine 30, no. 8: 1517–1519.

[fsn371808-bib-0019] Guo, H. L. , N. F. Dong , L. J. Hu , et al. 2017. “Recognition of Main Anti‐Dysmenorrhea Effect Components in Cyperi Rhizoma Based on Constituents Knock‐Out Strategy.” Chinese Journal of Experimental Traditional Medical Formulae 23, no. 10: 7–11.

[fsn371808-bib-0020] Guo, H. L. , J. C. Wang , L. J. Hu , et al. 2017. “The Comparison of the Anti‐Inflammatory and Analgesic Effects of Different Processed Products of Cyperus.” Journal of Jiangxi University of Chinese Medicine 29, no. 1: 74–83.

[fsn371808-bib-0021] He, G. , C. X. Wang , L. B. Zhang , et al. 2009. “Therapy of Zhuifeng Tougu Pills on Osteoarthritis With Chronic Kidney Disease.” Chinese Traditional Patent Medicine 31, no. 7: 991–993.

[fsn371808-bib-0022] He, J. C. , X. R. Li , and L. F. Yang . 2015. “Analysis of Volatile Constituents in Herbal Pair Artemisiae Argyi Folium‐Cyperi Rhizoma and Its Dingle Herbs.” Research and Practice on Chinese Medicines 29, no. 4: 37–40.

[fsn371808-bib-0023] Hu, L. J. , X. J. Zhao , H. L. Guo , et al. 2021. “Intestinal Absorption Mechanism of Cyperi Rhizoma With Jianchangbang by Single Pass Intestinal Perfusion Model in Rats.” China Journal of Traditional Chinese Medicine and Pharmacy 36, no. 3: 1392–1396.

[fsn371808-bib-0024] Hu, T. 2012. “58 Cases of Acute Jaundice Hepatitis Treated With Yueju Pills.” Chinese Medicine Modern Distance Education of China 10, no. 18: 15.

[fsn371808-bib-0025] Hu, Z. F. , L. J. Hu , H. L. Guo , et al. 2012. “Evaluation of Processing Technology for Jianchangbang *Cyperus rotundus* Based on Uniform Design Method.” Chinese Journal of Experimental Traditional Medical Formulae 18, no. 16: 39–41.

[fsn371808-bib-0026] Huang, B. X. , D. W. He , G. X. Chen , et al. 2018. “α‐Cyperone Inhibits LPS‐Induced Inflammation in BV‐2 Cells Through Activation of Akt/Nrf2/HO‐1 and Suppression of the NF‐κB Pathway.” Food & Function 9, no. 5: 2735–2743. 29667667 10.1039/c8fo00057c

[fsn371808-bib-0027] Huang, S. M. , and J. S. Qiu . 2017. “Clinical Study on Chaihu Shugan Powder Enema in the Treatment of Depression.” China's Naturopathy 25, no. 5: 32–33.

[fsn371808-bib-0028] Huang, Y. F. , Y. F. Tan , X. K. Ren , et al. 2023. “Mechanism of *Alpinia officinarum* ‐ *Cyperus rotundus* in the Treatment of Primary Dysmenorrhea Based on Network Pharmacology, Molecular Docking and Experimental Study.” Journal of Guangdong Pharmaceutical University 39, no. 6: 24–36.

[fsn371808-bib-0029] Hui, W. , and S. H. Sun . 2015. “Traditional Chinese Medicine Artificial Menstrual Cycle in Women With Premature Ovarian Failure a Randomized Controlled Study.” Journal of Practical Traditional Chinese Internal Medicine 29, no. 3: 22–25.

[fsn371808-bib-0030] Ji, N. P. , J. R. Lu , W. B. Li , et al. 2015. “Influences of Different Vinegar Processing Methods on Contents of Index Components in Cyperi Rhizoma.” Chinese Journal of Experimental Traditional Medical Formulae 21, no. 7: 5–7.

[fsn371808-bib-0031] Jia, M. R. 2007. Study on GAP of Production Quality Management Standard of Ligusticum Chuanxiong and Angelica Dahurica. Sichuan Science and Technology Press.

[fsn371808-bib-0033] Jin, Y. Q. , Y. L. Hong , J. R. Li , et al. 2013. “Advancements in the Chemical Constituents and Pharmacological Effects of Chuanxiong.” Pharmacology and Clinics of Chinese Materia Medica 4, no. 3: 44–48.

[fsn371808-bib-0034] Ke, L. , and X. H. Zhang . 2018. “Development Status, Existing Problems and Suggestions of Artemisia Argyi Industry in Xinxian County.” Agriculture of Henan 16: 16–17.

[fsn371808-bib-0035] Kilani, S. , R. B. Ammar , I. Bouhlel , et al. 2005. “Investigation of Extracts From (Tunisian) *Cyperus rotundus* as Antimutagens and Radical Scavengers.” Pharmacology 20, no. 3: 478–484. 10.1016/j.etap.2005.05.012 21783629

[fsn371808-bib-0036] Kilinc, E. , F. Tore , Y. Dagistan , and G. Bugdayci . 2020. “Thymoquinone Inhibits Neurogenic Inflammation Underlying Migraine Through Modulation of Calcitonin Gene‐Related Peptide Release and Stabilization of Meningeal Mast Cells in Glyceryltrinitrate‐Induced Migraine Model in Rats.” Inflammation 43, no. 1: 264–273. 31707574 10.1007/s10753-019-01115-w

[fsn371808-bib-0037] Lan, X. Y. , Y. Zhang , L. B. Zhu , et al. 2020. “Research Progress on Chemical Constituents From Artemisiae Argyi Folium and Their Pharmacological Activities and Quality Control.” China Journal of Chinese Materia Medica 45, no. 17: 4017–4030. 33164385 10.19540/j.cnki.cjcmm.20200714.201

[fsn371808-bib-0038] Li, B. , X. W. Liu , and F. B. Li . 2018. “Study on the Processing and Chemical Composition Changes of the Prescription of *Cyperus rotundus* .” Guizhou Medical Journal 42, no. 12: 1507–1508.

[fsn371808-bib-0040] Li, H. F. , Y. H. Li , Y. Wang , et al. 2014. “Advances in Studies on Chemical Constituents in Alpiniae Officinarum Rhizoma and Their Pharmacological Activities.” Chinese Journal of Experimental Traditional Medical Formulae 20, no. 7: 236–244.

[fsn371808-bib-0042] Li, Q. , P. Y. He , D. K. Zhang , et al. 2024. “Current Situation and Development Strategy of Chuanxiong Rhizoma Industry.” Chinese Traditional and Herbal Drugs 55, no. 8: 2771–2783.

[fsn371808-bib-0043] Li, R. L. , J. N. Zhou , X. Y. Zhang , et al. 2023. “Construction of the Gal‐NH2/Mulberry Leaf Polysaccharides‐Lysozyme/Luteolin Nanoparticles and the Amelioration Effects on Lipid Accumulation.” International Journal of Biological Macromolecules 253: 126780. 37699459 10.1016/j.ijbiomac.2023.126780

[fsn371808-bib-0046] Li, S. W. , and Z. F. Hu . 2013. “Effects of Vinegar Processed Rhizoma Cyperi on Rat Spinal c‐Fos Protein Expression.” Traditional Chinese Drug Research and Clinical Pharmacology 24, no. 2: 129–131.

[fsn371808-bib-0047] Li, S. X. , H. J. Lu , Y. X. Guo , et al. 2018. “GC‐MS Analysis of Chemical Constituents of the Essential Oil From Herbal Pair Tangerine Peel‐ *Cyperus rotundus* L.” Heilongjiang Medicine and Pharmacy 41, no. 4: 5–6.

[fsn371808-bib-0048] Li, W. B. , J. Li , L. Sheng , et al. 2025. “The Evolution of Ancient and Modern Processing Techniques of “Qizhi Xiangfu” and “Wuzhi Xiangfu” and Research on Modern Industrial Improvements.” Asia‐Pacific Traditional Medicine 21, no. 8: 51–58.

[fsn371808-bib-0049] Li, X. L. , Y. Eishi , Y. Q. Bai , et al. 2004. “Expression of the SRY‐Related HMG Box Protein SOX2 in Human Gastric Carcinoma.” International Journal of Oncology 24, no. 2: 257–263. 14719100

[fsn371808-bib-0052] Li, Y. X. , Y. S. Qu , and Q. Q. Ci . 2010. “Comparative Study on the Content of Total Saponins in *Cyperus rotundus* Before and After Processing.” Inner Mongolia Journal of Traditional Chinese Medicine 29, no. 11: 141–142.

[fsn371808-bib-0053] Li, Z. M. 2004. “Stomachache Treatment and Qi and Blood View.” Chinese Journal of Information on Traditional Chinese Medicine 11: 1024–1025.

[fsn371808-bib-0055] Li, Z. Z. , J. L. Lv , L. B. Zhang , et al. 2016. “Chemical Constituents and Pharmacology Activities of Artemisia Argyi:Research Advances.” Journal of International Pharmaceutical Research 43, no. 6: 1059–1066.

[fsn371808-bib-0056] Liang, G. P. , P. Cai , and L. Huang . 2018. “Analysis of the Differences Between the Components of “Jianchang Bang” and the Volatile Oil of Shengxiang.” World Latest Medicine Information 18, no. 99: 5–7.

[fsn371808-bib-0058] Liu, H. , M. L. Zhang , M. Yu , et al. 2020. “Antidepressant Activity Evaluation andGC‐MS Analysis of Volatile Oil From Vinegar‐Made Cyperi Rhizome.” Drug Evaluation Research 43, no. 3: 436–442.

[fsn371808-bib-0059] Liu, P. , E. X. Shang , Y. Zhu , D. W. Qian , and J. A. Duan . 2018. “Volatile Component Interaction Effects on Compatibility of Cyperi Rhizoma and Angelicae Sinensis Radix or Chuanxiong Rhizoma by UPLC‐MS/MS and Response Surface Analysis.” Journal of Pharmaceutical and Biomedical Analysis 160: 135–143. 30086506 10.1016/j.jpba.2018.07.060

[fsn371808-bib-0062] Ma, C. F. , J. B. Zhang , H. Y. Chen , et al. 2024. “Research Progress on Application of Chinese Herbal Medicine in Cattle Production.” Feed Research 47, no. 18: 163–167.

[fsn371808-bib-0063] Maes, M. , M. Kubera , J. C. Leunis , and M. Berk . 2012. “Increased IgA and IgM Responses Against Gut Commensals in Chronic Depression: Further Evidence for Increased Bacterial Translocation or Leaky Gut.” Journal of Affective Disorders 141, no. 1: 55–62. 22410503 10.1016/j.jad.2012.02.023

[fsn371808-bib-0064] Mao, X. P. , J. H. Mao , S. H. Zhou , et al. 1998. “Experimental Study on Compatibility of Folium Artemisiae Argyi and Rhizoma Cyperi.” Journal of Yunnan University of Chinese Medicine S1: 40–41.

[fsn371808-bib-0065] Misra, U. K. , J. Kalita , G. Tripathi , and S. K. Bhoi . 2017. “Role of β Endorphin in Pain Relief Following High Rate Repetitive Transcranial Magnetic Stimulation in Migraine.” Brain Stimulation 10, no. 3: 618–623. 28274721 10.1016/j.brs.2017.02.006

[fsn371808-bib-0066] Morales‐Payan, J. P. , B. M. Santos , W. M. Stall , and T. A. Bewick . 1998. “Interference of Purple Nutsedge ( *Cyperus rotundus* ) Population Densities on Bell Pepper ( *Capsicum annuum* ) Yield as Influenced by Nitrogen.” Weed Technology 12, no. 2: 230–234.

[fsn371808-bib-0067] Morales‐Payan, J. P. , W. M. Stall , D. G. Shilling , et al. 1997. “Effects of Purple Nutsedge ( *Cyperus rotundus* ) on Tomato ( *Lycopersicon esculentum* ) and Bell Pepper ( *Capsicum annuum* ) Vegetative Growth and Fruit Yield.” Weed Technology 11, no. 4: 672–676.

[fsn371808-bib-0069] Neamsuvan, O. , and T. Ruangrit . 2017. “A Survey of Herbal Weeds That Are Used to Treat Gastrointestinal Disorders From Southern Thailand: Krabi and Songkhla Provinces.” Journal of Ethnopharmacology 196: 84–93. 27956357 10.1016/j.jep.2016.11.033

[fsn371808-bib-0071] Niu, P. , T. Lan , J. Li , et al. 2021. “Effect of Chuanxiong Rhizoma‐Cyperi Rhizoma Combination on Migraine in a Murine Model.” Farmácia 69, no. 4: 771–777.

[fsn371808-bib-0072] Pan, K. , B. M. Gao , A. C. Wang , et al. 2016. “Analysis of Genetic Diversity of *Alpinia officinarum* Hance Revealed by ISSR Markers.” Molecular Plant Breeding 14, no. 8: 2224–2231.

[fsn371808-bib-0073] Park, A. J. , J. Collins , P. A. Blennerhassett , et al. 2013. “Altered Colonic Function and Microbiota Profile in a Mouse Model of Chronic Depression.” Neurogastroenterology and Motility 25, no. 9: 733‐e575. 23773726 10.1111/nmo.12153 PMC3912902

[fsn371808-bib-0075] Qiao, L. , Y. Y. Zhang , R. C. Wang , et al. 2022. “Effects of Different Processing Methods on Cyperotundone and α‐Cyperone in Xiangfu ( *Cyperus rotundus* L.).” Chinese Archives of Traditional Chinese Medicine 40, no. 1: 49–53.

[fsn371808-bib-0076] Qu, H. J. , K. W. Lin , X. L. Li , et al. 2021. “Chemical Constituents and Anti‐Gastric Ulcer Activity of Essential Oils of *Alpinia officinarum* (Zingiberaceae), *Cyperus rotundus* (Cyperaceae), and Their Herbal Pair.” Chemistry & Biodiversity 18, no. 10: e2100214. 34402190 10.1002/cbdv.202100214

[fsn371808-bib-0077] Rani, M. P. , and K. J. Padmakumari . 2012. “HPTLC and Reverse Phase HPLC Methods for the Simultaneous Quantification and In Vitro Screening of Antioxidant Potential of Isolated Sesquiterpenoids From the Rhizomes of *Cyperus rotundus* .” Journal of Chromatography. B, Analytical Technologies in the Biomedical and Life Sciences 904: 22–28. 22877740 10.1016/j.jchromb.2012.05.042

[fsn371808-bib-0078] Ren, L. , W. W. Tao , W. D. Xue , et al. 2015. “Potent Rapid Antidepressant Effects of Petroleum Ether Fraction of Yueju Pill Associated With BDNF and TrkB Up‐Regulation.” Chinese Pharmacological Bulletin 31, no. 12: 1754–1759.

[fsn371808-bib-0080] Rhodin, A. , A. Grönbladh , H. Ginya , et al. 2013. “Combined Analysis of Circulating β‐Endorphin With Gene Polymorphisms in OPRM1, CACNAD2 and ABCB1 Reveals Correlation With Pain, Opioid Sensitivity and Opioid‐Related Side Effects.” Molecular Brain 6: 8. 23402298 10.1186/1756-6606-6-8 PMC3602034

[fsn371808-bib-0081] She, J. M. , M. Zhong , Y. Z. Liang , et al. 2011. “Comparative Analysis of Volatile Constituents in Herbal Pair Ramulus Cinnamomi‐Atractylodes Macrocephala and Its Signal Herb by GC‐MS.” Journal of Central South University(Science and Technology) 42, no. 1: 22–27.

[fsn371808-bib-0083] Sheng, F. Y. , L. J. Zhou , X. Yan , et al. 2016. “Effects of *Cyperus rotundus* Before and After Vinegar Processing on Liver‐Qi Stagnation Model Rats.” Chinese Traditional Patent Medicine 38, no. 1: 156–159.

[fsn371808-bib-0086] Sun, S. J. , X. J. Han , R. P. Chi , et al. 2012. “Effects of Licorice and Hance Ginger Complex Extract on the Fresh‐Keeping of Spinach.” Food & Machinery 28, no. 3: 203–206.

[fsn371808-bib-0087] Sun, X. M. , Z. W. Zang , Y. Q. Cheng , et al. 2007. “Comparison of Pharmacological Experiments on Different Specifications of Xiangfu Decoction Pieces.” Journal of Chinese Medicinal Materials 10: 1219–1221.

[fsn371808-bib-0088] Sutalangka, C. , and J. Wattanathorn . 2017. “Neuroprotective and Cognitive‐Enhancing Effects of the Combined Extract of Cyperus Rotundus and *Zingiber officinale* .” BMC Complementary and Alternative Medicine 17, no. 1: 135. 28253877 10.1186/s12906-017-1632-4 PMC5335841

[fsn371808-bib-0089] Tang, C. Z. , C. G. Lv , L. Q. Huang , et al. 2020. “Pattern of Ecological Planting for Chinese Materia Medica Based on Regional Distribution.” China Journal of Chinese Materia Medica 45, no. 9: 1982–1989. 32495542 10.19540/j.cnki.cjcmm.20200302.103

[fsn371808-bib-0090] Thompson, C. A. , A. De‐La‐Forest , and M. A. Battle . 2018. “Patterning the Gastrointestinal Epithelium to Confer Regional‐Specific Functions.” Developmental Biology 435, no. 2: 97–108. 29339095 10.1016/j.ydbio.2018.01.006 PMC6615902

[fsn371808-bib-0092] Umerie, S. , and H. J. Ezeuzo . 2000. “Physicochemical Characterization and Utilization of *Cyperus rotundus* Starch.” Bioresource Technology 72, no. 2: 193–196.

[fsn371808-bib-0093] Wang, C. 2020. Study on Antifungual and Herbicidal Constituents From Tuber of *Cyperus rotundus* L. Guangxi University.

[fsn371808-bib-0095] Wang, H. B. , G. B. Feng , S. X. Li , et al. 2017. “Analysis of GC/MS on the Volatile Constituent Spectrum of Herb Pair Rhizoma Chuanxiong‐Rhizoma Cyperi.” Guangdong Chemical Industry 44, no. 7: 31–32.

[fsn371808-bib-0096] Wang, J. L. , Q. T. Guo , J. Zhang , et al. 2024. “Artemisia Argyi and Its Application in Traditional Chinese Medicine Agriculture.” Contemporary Horticulture 47, no. 17: 80–82.

[fsn371808-bib-0099] Wang, X. L. , D. N. Cao , and Z. Q. Shan . 2003. “Experimental Study of Aifu Nuangong Pill Treating Dysmenorrhea in Rats.” Journal of Hubei University of Chinese Medicine 2: 18–19.

[fsn371808-bib-0102] Wang, Y. , and L. H. Kasper . 2014. “The Role of Microbiome in Central Nervous System Disorders.” Brain, Behavior, and Immunity 38: 1–12. 24370461 10.1016/j.bbi.2013.12.015 PMC4062078

[fsn371808-bib-0103] Wang, Y. Z. , L. L. Wan , and K. Q. Li . 2009. “Study of the Different Extraction Methods in Compatibility With Gaoliangjiang and Xiangfu.” Journal of Liaoning University of TCM 11, no. 5: 182–183.

[fsn371808-bib-0104] Wei, X. H. , X. D. Xu , J. S. Shen , et al. 2009. “Anti‐Depressant Dffect of Yueju Ethanol Extract and Its Constituents in Mice Models of Despair.” China Pharmacy (Chongqing, China) 20, no. 3: 166–168.

[fsn371808-bib-0105] Wu, S. , T. Zhao , L. Jin , and M. Gong . 2024. “Exploring the Synergistic Effects of Chuanxiong Rhizoma and Cyperi Rhizoma in Eliciting a Rapid Anti‐Migraine Action Based on Pharmacodynamics and Pharmacokinetics.” Journal of Ethnopharmacology 335: 118608. 39053709 10.1016/j.jep.2024.118608

[fsn371808-bib-0107] Xu, J. , W. X. Zeng , X. D. Wang , et al. 2022. “Research Progress on Chemical Constituents and Pharmacological Effects of Tangerine Peel.” Chinese Wild Plant Resources 41, no. 10: 72–106.

[fsn371808-bib-0108] Xue, M. J. , Y. Sun , and M. Huang . 2020. “Efficacy of Modified Xiangsu Powder in Dialectical Adjuvant Therapy for Functional Dyspepsia and Its Influenceon Gastric Motility.” Journal of Sichuan of Traditional Chinese Medicine 38, no. 5: 108–111.

[fsn371808-bib-0109] Xue, W. , X. Zhou , N. Yi , et al. 2013. “Yueju Pill Rapidly Induces Antidepressant‐Like Effects and Acutely Enhances BDNF Expression in Mouse Brain.” Evidence‐Based Complementary and Alternative Medicine 2013: 184367. 23710213 10.1155/2013/184367 PMC3654702

[fsn371808-bib-0110] Yan, X. A. , and M. A. Lin . 2003. “Preparation of Volatile Oil From Rhizoma Alpiniae Officinarum and Its Application in Tobacco Flavoring.” Tobacco Science and Technology 12: 11–12.

[fsn371808-bib-0112] Yang, L. J. 2013. Study on the Material Basis of Chuanxiong Rhizoma‐Cyperi Rhizoma Effect In Vivo. Xi Bei University.

[fsn371808-bib-0113] Yang, Q. , C. Zhang , H. Chen , et al. 2011. “AFLP Analysis of Genetic Diversity of *Alpinia officinarum* .” China Journal of Chinese Materia Medica 36, no. 3: 330–333. 21585037

[fsn371808-bib-0114] Yang, T. G. , S. T. Ni , X. H. Gao , et al. 2021. “Volatile Components in Cyperi Rhizome Prepared With Vinegar by HS‐GC‐MS.” Central South Pharmacy 19, no. 5: 865–869.

[fsn371808-bib-0115] Ye, X. 2008. “Mixing Mesotrione With Atrazine or Bentazone to Control Cyperus Esculentus and *Cyperus rotundus* in Corn Field.” World Pesticide 30, no. 6: 47.

[fsn371808-bib-0116] Yong, D. Q. , and M. L. Feng . 2000. “Effects of Compatibility of Radix Astragali Seu Hedysari, Radix Angelicae Sinensis, Rhizoma Cyperi, Rhizoma Chuanxiong and Radix Paeoniae Rubra on Hemorheology in Normal Rats.” Journal of Pharmaceutical Research 2: 29–30.

[fsn371808-bib-0117] Yu, M. , H. M. Jia , H. W. Zhang , et al. 2020. “The Regulatory Effects of Chaihu‐Shu‐Gan‐San on Fecal Metabolome and Gut Microbiota in Depression Model Rats.” Journal of International Pharmaceutical Research 47, no. 3: 229–235.

[fsn371808-bib-0119] Zeng, C. , and A. Z. Yang . 1991. “Xiangfu Decoction in the Treatment of 36 Cases of Mastitis.” Hainan Medical Journal 3: 38–39.

[fsn371808-bib-0120] Zhang, H. F. 2004. “Guishao Dihuang Decoction Combined With Yueju Pill in the Treatment of 60 Cases of Polycystic Ovary Syndrome.” Journal of Practical Traditional Chinese Medicine 2: 70.

[fsn371808-bib-0121] Zhang, L. L. , L. F. Zhang , Q. P. Hu , D. L. Hao , and J. G. Xu . 2017. “Chemical Composition, Antibacterial Activity of *Cyperus rotundus* Rhizomes Essential Oil Against *Staphylococcus aureus* via Membrane Disruption and Apoptosis Pathway.” Food Control 80: 290–296.

[fsn371808-bib-0123] Zhang, S. J. , J. B. Guo , X. L. Wang , et al. 2013. “The Pharmacological Actions of the Extracts From Chuanxiong Rhizoma and Cyperi Rhizoma on Dysmenorrhea Models in Murine.” Journal of Shenyang Pharmaceutical University 30, no. 5: 383–387.

[fsn371808-bib-0124] Zhang, W. J. , Y. Xu , and Q. W. Wang . 2011. “Analysis of the Diversify in the Matched Pair of Chuanxiong Rhizoma and Cyperi Rhizoma by HPLC.” China Medical Herald 8, no. 20: 96–100.

[fsn371808-bib-0125] Zhang, X. Y. 2015. Study on the Extraction and the Pharmacokinetics in Rat of theRhizoma Chuanxiong‐Rhizoma Cyperi Herb Couple. Air Force Medical University of Chinese PLA.

[fsn371808-bib-0126] Zhang, X. Y. , Q. W. Wang , W. J. Zhang , et al. 2013. “Content Change of Main Component of Extracts of Different Proportions ofLigusticum Wallichii‐Rhizoma Cyperi.” China Medical Herald 10, no. 19: 121–123.

[fsn371808-bib-0127] Zhang, Y. , Y. H. Feng , H. Zhang , et al. 2011. “60 Cases of Chronic Pharyngitis Treated With Yueju Pill.” Chinese Journal of Experimental Traditional Medical Formulae 17, no. 10: 297.

[fsn371808-bib-0128] Zhang, Y. X. , B. Cui , Z. L. Zou , et al. 2018. “Clinical Observation on Effect of Yueju Pill in Treating Qi‐Stagnation Constitution Depression Patients.” Liaoning Journal of Traditional Chinese Medicine 45, no. 5: 960–963.

[fsn371808-bib-0129] Zhang, Z. 2014. “Extraction and Separation Skin‐Whitening Agentfrom Rhizoma Chuanxiong and Its Application Incosmetics.” Guangdong Yao Xue Yuan 2014: 1–67.

[fsn371808-bib-0130] Zhao, X. J. , L. J. Hu , and H. L. Guo . 2018. “Study on the Material Basis of Regulating Menstruation and Relieving Pain of Four Processed *Cyperus rotundus* .” Yunnan Journal of Traditional Chinese Medicine and Materia Medica 39, no. 9: 73–75.

[fsn371808-bib-0131] Zhou, L. J. , X. Yan , N. P. Ji , et al. 2016. “Effects of Different Extracts of *Cyperus rotundus* Processed With Vinegar on Gastrointestinal Motility in Rats With Gastrointestinal Dysfunction of Liver Depression Type.” Journal of Chinese Medicinal Materials 39, no. 1: 174–177.

[fsn371808-bib-0133] Zhou, Y. C. , F. L. Zhao , J. H. Chen , et al. 2002. “Effect of Kaixin Capsule in Reconstructing the Collagen of Non‐Infarct Region of Left Ventricle in Rats After Myocardial Infarction.” Journal of Guangzhou University of Traditional Chinese Medicine 3: 204–206.

