# Supplementary material for: Unlocking the Bioactivity of Cyperi Rhizoma as a Functional Food: Insights From Culinary Processing and Herb Combinations
Source: Food Sci Nutr. 2026 May 15;14(5):e71808. doi: 10.1002/fsn3.71808 (PMC13178274; doi:10.1002/fsn3.71808)
Supplement: Supplementary file 1 — Figure S1: Schematic diagram of CZ plant. Figure S2: Schematic diagram of CH plant. Figure S3: PPI and H‐C‐T network analysis of 44 potential therapeutic targets for CXP in HCC (Qing et al. 2022). Table S1: Clinical application of CR‐CX herb pair. Table S2: Clinical application of CR‐GLJ herb pair. Table S3: Clinical application of CR‐AY herb pair. Table S4: Clinical application of CR‐CP herb pair. Table S5: The main chemical composition of CR‐CZ herb pair. Table S6: Clinical application of CR‐CZ herb pair. Table S7: Clinical application of CR‐CH herb pair. [file FSN3-14-e71808-s001.docx]

**Unlocking the Bioactivity of Cyperi Rhizoma as a Functional Food: Insights from Culinary Processing and Herb Combinations**

**Yuehan Liu^1△^ Liyuan Xu^2,3,4△^ Liting Lin^2,3,4^ Maoyuan Jiang^2,3,4^ Wan Liao^2,3,4*^ Tianhui Gao^1*^**

**^1^** School of Pharmacy**, Qilu Medical University, Zibo, Shandong 255000, China**

^2^ School of Pharmacy, Chengdu University of Traditional Chinese Medicine, Chengdu, Sichuan 611137, China.

^3^ Key Laboratory of Standardization of Chinese Medicine (Chengdu University of Traditional Chinese Medicine), Ministry of Education

^4^ Lab for Innovation & Effective Uses of Chinese Drug Germplasm Resources

**^△^** These authors made equal contributions to this work.

^*^ Correspondence author: liaowan@cdutcm.edu.cn (W. Liao); gaotianhui@qlmu.edu.cn (TH. Gao)

**TABLE S1 |** Clinical application of CR-CX herb pair

| **NO.** | **Clinical data** | | | | | **Ref.** |
| --- | --- | --- | --- | --- | --- | --- |
|  | **Diseases** | **Cases** | **Medicinal** | **Dosing** | **Results** |  |
| 1 | Coronary heart disease caused stable angina pectoris complicated with anxiety | 30 cases,14 males and 16 females, aged 50 to 70, with a history of coronary heart disease spanning a duration of 1 to 10 years. | **CR 15 g, CX 12g,** CZ 10g, Massa Medicata Fermentata (Shenqu)10g, Amomi Fructus (Sharen) 10g, Salviae Miltiorrhizae Radix et Rhizoma (Danshen) 15g, Gardeniae Fructus (Zhizi) 6g, Santali Albi Lignum (Tanxiang) 6g. | 4 weeks. | Total effective rate: 83.33%  Cure rate:6.67% | (Wang and Yi 2023) |
| 2 | Refractory gastroesophageal reflux disease | 30 cases, 19 males and 11 females, and aged 26 to 64. | **CR 15 g, CX 10g,** Caryophylli Flos (Dingxiang) 10g, Kaki Calyx (Shidi) 15g, CP 15g, Citri Reticulatae Pericarpium Viride (Qingpi) 12g, Massa medicata fermentata (Shenqu)12g, Perillae Caulis (Zisugeng)12g, Ginseng Radix et Rhizoma (Renshen) 9g, CZ 9g, Gardeniae Fructus Praeparatus (Jiaozhizi) 9g. | 8 weeks. | Total effective rate: 86.67% | (Li 2022) |
| 3 | Diabetes | 30 cases,17 males and 13 females, aged 30 to 70. | **CR 15 g, CX 10g,** Gardeniae Fructus (Zhizi) 6g, CZ 10g, Massa Medicata Fermentata (Shenqu) 10g, Scrophulariae Radix (Xuanshen) 10g, Coptidis Rhizoma (Huanglian) 6g. | 8 weeks. | Total effective rate: 83.33%  Cure rate:16.67% | (Chen et al. 2020) |
| 4 | Perimenopausal insomnia | 36 cases, age 48 to 55 years old. The maximum duration of the disease is 12 months, with a minimum of 3 months. | **CR 10 g, CX 6g,** Lilii Bulbus (Baihe) 15g, Rehmanniae Radix (Shengdihuang) 15g, Draconis Dens (qinglongchi) 15g, Gardeniae Fructus Praeparatus (Jiaozhizi) 10g, Massa Medicata Fermentata (Shenqu)10g, CZ 10g, Ziziphi Spinosae Semen (Suanzaoren)20g, Polygoni Multiflori Caulis (Shouwuteng) 30g, Polygalae Radix (Yuanzhi) 6g, Anemarrhenae Rhizoma (Zhimu) 10g, Phellodendri Chinensis Cortex (Huangbo) 10g, Lycii Cortex (Digupi) 10g, CH 10g, Curcumae Radix (Yujin) 10g, Salviae Miltiorrhizae Radix et Rhizoma (Danshen) 15g, Triticum aestivum L. (Huaixiaomai) 30g. | 4 weeks. | Total effective rate: 94.4%  Cure rate:25% | (Zhang and Xie 2015) |
| 5 | Hyperlipidemia | 44 cases, 23 males and 21 females, aged 41 to 79 years old.Disease duration 1-16 years; 16 cases with elevated total cholesterol (TC) alone, 15 cases with elevated triacylglycerol (TG) alone, 13 cases with both elevated TC and TG, and 14 cases with reduced High-density lipoprotein cholesterol (HDL-C) | **CR 12 g, CX 12g,** CZ 12g, Massa Medicata Fermentata (Shenqu)12g, Gardeniae Fructus Praeparatus (Chaozhizi) 12g, Gastrodiae Rhizoma (Tianma) 12g, Trichosanthis Fructus (Gualou) 30g, Salviae Miltiorrhizae Radix Rhizoma (Danshen) 30g, Lycii Fructus (Gouqizi) 12g. | 8 weeks. | Total effective rate: 79.55% | (Sun 2008) |
| 6 | Functional dyspepsia | 60 cases,26 males and 34 females, aged 24 to 67 years old. The disease duration ranged from six weeks to five years, with all patients experiencing persistent or recurrent epigastric discomfort. | **CR, CX,** CZ, Gardeniae Fructus (Zhizi), Massa Medicata Fermentata (Shenqu), from 10g to 12g each, should be added or subtracted by the requisite specifications. | 1 month. | Total effective rate: 88.3%  Cure rate:41.7% | (Shi 2005) |
| 7 | Gastrointestinal functional disorder | Male, 45 years old. Symptoms: The patient presents with a conscious awareness of abdominal discomfort, which is often distended and exhibits a lack of fixed location. The pain is further exacerbated by irritation. | **CR 15g, CX 10g,** CZ 10g，Massa Medicata Fermentata (Shenqu)10g, Gardeniae Fructus Praeparatus (Jiaozhizi) 10g, Arecae Semen (Binglang) 10g, CH 10g, Paeoniae Radix Alba (Baishao) 10g, Linderae Radix  (Wuyao) 10g, Pseudostellariae Radix (Taizishen) 5g, Glycyrrhizae Radix et Rhizoma (Gancao) 5g, Aquilariae Lignum Resinatum (Chenxiang) 3g. | 21 doses. | Heal | (Qi 2014) |
| 8 | Coronary angina pectoris | 60 cases, 30 cases in the control group, 19 males and 11 females, age 45 to 78 years,13 cases with hypertension, 4 cases with diabetes, 2 cases with arrhythmia, degree of angina pectoris: 10 cases of mild, 17 cases of moderate, 3 cases of severe.; 30 cases in the study group,18 males and 12 females， age 47 to 76 years,11 cases with hypertension, 3 cases with diabetes, 3 cases with arrhythmia, degree of angina pectoris: 19 cases of mild, 8 cases of moderate, 3 cases of severe. | Control grpup: Oral Isosorbide Dinitrate (Xiaoxintong).  Study group: **CR 15g, CX 12g,** CZ 10g, Massa Medicata Fermentata (Shenqu)10g, Gardeniae Fructus (Zhizi) 6g and add or subtract medicines according to the symptoms. | 1 month. | Total effective rate: 55.00%(control group);66.67%(study group) | (Shen 2007) |
| 9 | Gastric neurosis | Female，35 years old. The symptoms include abdominal discomfort, intermittent pain, loss of appetite, and a tendency to become irritated with minimal provocation. | **CR 15g, CX 15g,** CZ 15g, Massa Medicata Fermentata (Shenqu) 15g, Gardeniae Fructus (Zhizi) 15g, Curcumae Radix (Yujin) 15g, Aurantii Fructus (Zhiqiao) 15g, Magnoliae Officinalis Cortex (Houpo) 15g, CH 10g, Glycyrrhizae Radix et Rhizoma (Gancao)10g, Amomi Fructus Rotundus (Doukou) 10g. | 7 doses. | Heal | (Mou 2009) |
| 10 | Antipsychotic-induced hyperprolactinemia | 46 cases, age 17 to 55 years old. | **CR 12g ,CX 9g,** CZ 12g, Gardeniae Fructus (Zhizi)12g, Acori Tatarinowii Rhizoma (Shichangpu) 12g， Curcumae Radix (Yujin) 12g, Glycyrrhizae Radix et Rhizoma (Gancao) 12g, Hordei Fructus Germinatus (Maiya) 100g. | 2 months. | Total effective rate: 73.9% | (Tong et al. 2007) |
| 11 | Chronic gastritis | 60 cases, 38 males and 22 females. Age 17-74 years. Year old.  36 cases of chronic superficial gastritis, 8 cases of chronic atrophic gastritis, 16 cases of compound gastritis, and 43 cases were HP positive. | **CR in vinger 15g , CX 12g,** CZ 10g, Massa Medicata Fermentata (Shenqu)15g, Gardeniae Fructus Praeparatus (Jiaozhizi) 10g, CP 6g, Arecae Semen (Binglang) 10g, Pinelliae Rhizoma Praeparatum Cum Zingibere et Alumine (Jiangbanxia) 10g, Glycyrrhizae Radix et Rhizoma (Gancao)  6g and add or subtract medicines according to the symptoms. | 3 months. | Total effective rate: 98.3%  Cure rate:53.3% | (Wang and Zhao 2000) |
| 12 | Upper digestive tract ulcer | 111 cases, 105 males and 6 females. Age 9 to 65 years old. Duodenal ulcer 59 cases, gastric ulcer 52 cases, duration of disease less than one year 61 cases, 3 years 34 cases, more than three years 16 cases | **CR 15g, CX 15g,** Gardeniae Fructus (Zhizi) 10g, Massa Medicata Fermentata (Shenqu)15g, Atractylodis Macrocephalae Rhizoma (Baizhu) 10g, Cleistocactus sepium (Wuzeigu) 10g, Corydalis Rhizoma (Yanhusuo) 20g, Bletillae Rhizoma (baiji) 2g, and add or subtract medicines according to the symptoms. | 1 to 3 months. | Total effective rate: 95% (Duodenal ulcer)  Cure rate: 61% (Duodenal ulcer); 88% (Gastric ulcer) | (Cheng et al. 1995) |
| 13 | HBV hepatitis | 36 cases, 18 cases in the control group, 11 males and 7 females, age 23 to 68 years old;18 cases in the study group,10 males and 8 females, age 23 to 68 years old. | **CR 10g, CX 10g,** CZ 15g, Massa Medicata Fermentata (Shenqu)10g, Gardeniae Fructus (Zhizi) 10g (Control group); CR 10g, CX 10g, CZ 15g, Massa Medicata Fermentata (Shenqu)10g, Gardeniae Fructus (Zhizi) 10g, Citri Exocarpium Rubrum (Juhong) 10g, Atractylodis Macrocephalae Rhizoma (Baizhu) 10g, Scutellariae Radix (Huangqin) 10g, Crataegi Fructus (Shanzha) 10g (Study group). | 3 months. | Total effective rate: 76.92% (Control group);  94.23% (Study group); | (Wang 2017) |
| 14 | Alcoholic fatty liver | 60 cases, 49 males and 11 females. Age 26-40 years old 48 cases, over 41 years old 12 cases. The disease was mild in 25 cases, moderate in 29 cases, and severe in 6 cases. There were 29 cases of moderate disease and 6 cases of severe disease. | **CR 15g, CX 12g,** CZ 12g, Gardeniae Fructus (Zhizi) 15g, Rhei Radix et Rhizoma (Dahuang) 6g, CH 15g, Crataegi Fructus (Shanzha)30g, Alismatis Rhizoma (Zexie) 15g. | 1 to 2 months. | Total effective rate: 85%. | (Liu and Yan 2013) |
| 15 | Acute jaundice hepatitis | 58 cases, 43 males and 15 males, aged 12 to 59 years old. Serum total bilirubin was higher than 35 μmol/L, serum-conjugated bilirubin was higher than 18 μmol/L, and alanine aminotransferase and glutamine aminotransferase were higher than 40 kamagens. | **CR 10g, CX 10g,** CZ 10g, Massa Medicata Fermentata (Shenqu) 10g, Gardeniae Fructus (Zhizi) 10g, Artemisiae Scopariae Herba (Yinchen) 15g, Coicis Semen (Yiyiren) 15g, Poria (Fuling) 15g, Isatidis Radix (Banlangen) 15g, Plantaginis Semen (Cheqianzi) 15g, Toosendan Fructus (Chuanlianzi) 12g, Galli Gigerii Endothelium Corneum (Jineijin) 12g, Schisandrae Chinensis Fructus (Wuweizi) 5g, Amomi Fructus (Sharen) 5g. | 5 to 10 doses. | Total effective rate: 98.2%. | (Hu 2012) |
| 16 | Nervous headache | 41 cases, 10 males and 31 females, aged 15 to 40 years old. The shortest duration of the disease is six months, and the longest is 20 years. | **CR 10g, CX 15g,** Massa Medicata Fermentata (Shenqu) 20g, Cassiae Semen (Juemingzi) 20g, Tribuli Fructus (Jili)20g, Paeoniae Radix Alba (Baishao) 20g, Uncariae Ramulus Cum Uncis (Gouteng) 15g, Prunellae Spica (Xiakucao) 12g, CZ 10g, Gardeniae Fructus (Zhizi) 10g, Chrysanthemi Flos (Juhua) 10g, Glycyrrhizae Radix et Rhizoma (Gancao) 6g, Scorpio (Quanxie) 6g, Scolopendra (Wugong) 6g and add or subtract medicines according to the symptoms. | 7 to 28 days. | Total effective rate: 100%  Cure rate: 73.17% | (Wang 2010) |
| 17 | Dysmenorrhea | Female, 26 years old. Dysmenorrhea, scanty menstrual flow, dark red color with blood clots | **CR 20g, CX 10g,** CZ 10g, Massa Medicata Fermentata (Shenqu) 15g, Gardeniae Fructus (Zhizi) 10g, Angelicae Sinensis Radix (Danggui) 12g, Persicae Semen (Taoren) 12g, Carthami Flos (Honghua) 10g, Trogopterori Faeces (Wulingzhi) 15g, Salviae Miltiorrhizae Radix et Rhizoma (Danshen) 10g, Corydalis Rhizoma (Yanhusuo) 10g. | 3 months. | Heal. | (Cui et al. 2009) |
| 18 | Irregular menstruation | Female,25 years old. Irregular menstruation for 3 years, sometimes late or early, irregular, scanty, thick, purplish-black with blood clots. | **CR 15g, CX 12g,** CZ 10g, Massa Medicata Fermentata (Shenqu) 8g, Gardeniae Fructus (Zhizi) 10g, Angelicae Sinensis Radix (Danggui) 10g, Paeoniae Radix Alba (Baishao) 8g, Leonuri Herba (Yimucao) 10g, Rehmanniae Radix (Dihuang) 10g, Persicae Semen (Taoren) 10g, Curcumae Radix (Yujin) 10g, Toosendan Fructus (Chuanlianzi) 8g, Albiziae Flos (Hehuanhua) 10g, Aurantii Fructus (Zhiqiao) 8g. | 6 months. | Heal. | (Cui et al. 2009) |
| 19 | Menopause | Female, 30 years old. Two months after menopause, characterized by lower abdominal distension and pain, upset and irritable | **CR 12g, CX 10g,** Gardeniae Fructus (Zhizi) 10g, Aurantii Fructus (Zhiqiao) 10g. Corydalis Rhizoma (Yanhusuo) 10g, CZ 10g, Carthami Flos (Honghua) 10g, Massa Medicata Fermentata (Shenqu) 15g, Liquidambaris Fructus (Lulutong) 12g, Cyathulae Radix (Chuanniuxi) 15g, Lycopi Herba (Zelan) 12g. | 16 doses. | Heal. | (Cui et al. 2009) |
| 20 | Chronic pharyngitis | 60 cases, 21 males and 39 females, aged 23 to 64 years old. | **CR 15g, CX 15g,** CZ 15g, Massa Medicata Fermentata (Shenqu) 15g, Gardeniae Fructus (Zhizi) 15g, and add or subtract medicines according to the symptoms. | 21 doses. | Total effective rate: 71.6% (males);  84.6%(females) | (Zhang et al. 2011) |
| 21 | Paraesthesia pharyngis | 50 cases, 16 males and 34 females, aged 17 to 70 years old. All of them have different degrees of pharyngeal foreign body sensation, obstruction, neck tightness, girdle sensation, or choking sensation.  Tightness in the neck, aggravated by empty pharynx or emotional stimulation, mental stress. | **CR 10g, CX 6g,** Massa Medicata Fermentata (Shenqu) 10g, Gardeniae Fructus (Zhizi) 6g, CZ 6g, Pinelliae Rhizoma (Banxia) 10g, Perillae Caulis (Zisugeng) 10g, Tetrapanacis Medulla (Tongcao) 10g, Clematidis Radix et Rhizoma (Weilingxian) 10g, Acori Tatarinowii Rhizoma (Shichangpu) 10g, Citri Sarcodactylis Fructus (Foshou) 10g, Inulae Flos (Xuanfuhua) 10g and add or subtract medicines according to the symptoms. | 10 to 15 doses | Total effective rate: 92% | (Ren 2010) |

**TABLE S2 |** Clinical application of CR-GLJ herb pair

| **NO.** | **Clinical data** | | | | | **Ref.** |
| --- | --- | --- | --- | --- | --- | --- |
|  | **Diseases** | **Cases** | **Medicinal** | **Dosing cycles** | **Results** |  |
| 1 | Chronic gastritis | Female, 55 years old. | **CR 10g, GLJ 8g,** Lilii Bulbus (Baihe) 15 g, Linderae Radix (Wuyao) 10 g, Salviae Miltiorrhizae Radix et Rhizoma (Danshen) 15 g, Santali Albi Lignum (Tanxiang) 6 g, Carthami Flos (Honghua) 6 g, Amomi Fructus (Sharen) 6 g, CP 10 g, Perillae Caulis (Sugeng) 10 g, Citri Sarcodactylis Fructus (Foshou) 20 g, Aconiti Lateralis Radix Praeparata (Fuzi) 8 g, Zingiberis Rhizoma (Ganjiang) 10 g, Magnoliae Officinalis Cortex (Houpo) 10 g, Aurantii Fructus (Zhiqiao) 10 g | 29 doses | Heal | (Li et al. 2015) |
| 2 | Chronic superficial gastritis | 56 cases, 36 males, 20 females; 18 cases were aged 22-40 years and 38 cases were aged 41-70 years; 35 cases had a disease duration of 1-10 years and 21 cases had a disease duration of more than 10 years. | **CR 15g, GLJ 12g,** CH 12 g, Aurantii Fructus Immaturus (Zhishi) 12 g, Paeoniae Radix Alba (Baishao) 15 g, Glycyrrhizae Radix et Rhizoma Praeparata cum Melle (Zhigancao) 6 g, Poria (Fuling) 15 g, Atractylodis Macrocephalae Rhizoma Praeparatum (Chaobaizhu) 12 g, Pinelliae Rhizoma Praeparatum (Zhibanxia) 12 g, Aucklandiae Radix (Muxiang) 12 g, Galli Gigerii Endothelium Corneum (Jineijin) 15 g, Hordei Fructus Germinatus (Maiya) 15 g | 8 weeks | Total effective rate: 94.6% | (Zhou and Xiang 2012) |
| 3 | Peptic ulcer | 60 cases, 40 males, 20 females, age 17 to 75 years, duration of disease 28 days to 21 years. | **CR 10g, GLJ 5g,** Cinnamomi Ramulus (Guizhi) 10 g, Euodiae Fructus (Wuzhuyu) 3 g, Pinelliae Rhizoma (Banxia) 10 g, CP 10 g, Paeoniae Radix Alba (Baishao) 15 g, Massa Medicata Fermentata (Shenqu) 15 g, Galli Gigerii Endothelium Corneum (Jineijin) 10 g, Glycyrrhizae Radix et Rhizoma (Gancao) 3 g | 3 weeks | Total effective rate: 91.7% | (Gu 2008) |
| 4 | Chronic cholecystitis | 58 cases, 32 males, 26 females, age 32 to 68. | **CR 15g, GLJ 20g,** Curcumae Radix (Yujin) 30 g, CH 20 g, Paeoniae Radix Rubra (Chishao) 18 g, Paeoniae Radix Alba (Baishao) 18 g, Salviae Miltiorrhizae Radix et Rhizoma (Danshen) 25 g, Citri Reticulatae Pericarpium Viride (Qingpi) 15 g, CP 15 g, Cinnamomi Cortex (Guangui) 10 g, Euodiae Fructus (Wuzhuyu) 10 g, Raphani Semen (Laifuzi) 15 g, CZ 15 g, Atractylodis Macrocephalae Rhizoma (Baizhu) 15 g, Magnoliae Officinalis Cortex (Houpo) 20 g, Citri Fructus (Xiangyuan) 15 g | - | Total effective rate: 94.8% | (Jia 1996) |
| 5 | Bile reflux gastritis | 40 cases, 36 males, 4 females, age from 18 to 56, duration of disease from 1month to 20 years | **CR 10 g, GLJ 20g,** Codonopsis Radix (Dangshen) 20 g, Astragali Radix (Huangqi) 25 g, Curcumae Rhizoma (Ezhu) 10 g, CH 15 g, Pinelliae Rhizoma (Banxia) 15 g, Euodiae Fructus (Wuzhuyu) 5 g, Poria (Fuling) 20 g, Inulae Flos (Xuanfuhua) 20 g, Curcumae Radix (Yujin) 20 g, Corydalis Rhizoma (Yuanhu) 10 g, Aurantii Fructus (Zhiqiao) 15 g, Paeoniae Radix Alba (Baishao) 25 g, Glycyrrhizae Radix et Rhizoma (Gancao) 10 g | 35 days | Total effective rate: 95% | (Li and Liu 1985) |

“-” indicates that it was not mentioned in the original reference.

**TABLE S3 | Clinical application of CR-AY herb pair**

| **NO.** | **Clinical data** | | | | | **Ref.** |
| --- | --- | --- | --- | --- | --- | --- |
|  | **Diseases** | **Cases** | **Medicinal** | **Dosing cycles** | **Results** |  |
| 1 | Dysmenorrhea | 29 cases, aged 16 to 33 years | **CR 10g, AY10g,** Euodiae Fructus (Wuzhuyu) 12 g, Cinnamomi Cortex (Rougui) 10 g, Astragali Radix (Huangqi) 20 g, Angelicae Sinensis Radix (Danggui) 15 g, Rehmanniae Radix (Shengdihuang) 10 g, Paeoniae Radix Alba (Baishao) 10 g, CX 10 g, Dipsaci Radix (Xuduan) 10 g | 3 months | Total effective rate:75.9% | (Wang 2015) |
| 2 | Benign prostatic hyperplasia | 44 cases, aged 51 to 75 years, average (62.6±13.7) years; disease duration 3-11 years, average (6.4±4.8) years. | **CR 6g, AY9g,** Dipsaci Radix (Xuduan) 9 g, Paeoniae Radix Alba (Baishao) 9 g, Angelicae Sinensis Radix (Danggui) 12 g, CX 6 g, Euodiae Fructus (Wuzhuyu) 6 g, Cinnamomi Cortex (Rougui) 6 g, Rehmanniae Radix Praeparata (Shudihuang) 15 g, Astragali Radix (Huangqi) 15 g | 2 months | Total effective rate: 100% | (Li and Liu 2015) |
| 3 | Functional dysfunctional uterine bleeding | 63 cases, aged 15 to 45 years. | **CR 12g, AY6g,** Euodiae Fructus (Wuzhuyu) 6 g, CX 6 g, Paeoniae Radix Alba (Baishao) 6 g, Astragali Radix (Huangqi) 6 g, Dipsaci Radix (Xuduan) 5 g, Rehmanniae Radix (Shengdihuang) 6 g, Cinnamomi Cortex (Guangui) 5 g, Angelicae Sinensis Radix (Chuangui) 6 g | 1-3 months | Total effective rate: 87.3% | (Li and He 2013) |
| 4 | Dysmenorrhea | 91 cases, aged 14 to 39 years, disease duration 1 month to 24 years. | **CR 18g, AY15g,** Euodiae Fructus (Wuzhuyu) 12 g, CX 15 g, Paeoniae Radix Alba (Baishao) 15 g, Angelicae Sinensis Radix (Danggui) 15 g, Astragali Radix (Huangqi) 15 g, Dipsaci Radix (Xuduan) 15 g, Rehmanniae Radix (Shengdihuang) 9 g, Cinnamomi Cortex (Rougui) 6 g | 9 doses | Total effective rate: 92.31% | (Guo and He 2011) |

**TABLE S4 | Clinical application of CR-CP herb pair**

| **NO.** | **Clinical data** | | | | | **Ref.** |
| --- | --- | --- | --- | --- | --- | --- |
|  | **Diseases** | **Cases** | **Medicinal** | **Dosing cycles** | **Results** |  |
| 1 | Functional dyspepsia | 98 cases | **CR 10g, CP 10g,** Codonopsis Radix (Dangshen) 15 g, Atractylodis Macrocephalae Rhizoma Praeparatum (Chaobaizhu) 15 g, CH 10 g, Scutellariae Radix (Huangqin) 6 g, Poria Pararadicis (Fushen) 10 g, Nelumbinis Semen (Lianzi) 10 g, Albiziae Flos (Hehuanhua) 10 g, Jujubae Fructus (Dazao) 4 pieces, Glycyrrhizae Radix et Rhizoma Praeparata cum Melle (Zhigancao) 6 g | 4 weeks | Total effective rate: 90.81%  Cure rate: 61.22% | (Lu 2015) |
| 2 | Hyperplasia of mammary glands | 47 cases, aged 22 to 54 years, with disease durations ranging from 6 months to 10 years. | **CR 15g, CP 15g,** CH 15 g, CX 15 g, Aurantii Fructus (Zhiqiao) 15 g, Magnoliae Officinalis Cortex (Houpo) 15 g, Paeoniae Radix Alba (Baishao) 15 g, Pinelliae Rhizoma Praeparatum Cum Alumine (Qingbanxia) 10 g, Sparganii Rhizoma (Sanleng) 10 g, Curcumae Rhizoma (Ezhu) 15 g, Trichosanthis Pericarpium (Gualoupi) 15 g, Glycyrrhizae Radix et Rhizoma (Gancao) 10 g. | 3 to 5 months | Total effective rate: 93.62% | (Yang and Yan 2013) |
| 3 | Constipation | Female, 48 years old. | **CR 15g, CP 6g,** CH 10 g, Paeoniae Radix Alba (Baishao) 20 g, Aurantii Fructus (Zhiqiao) 15 g, Glycyrrhizae Radix et Rhizoma (Gancao) 6 g, CX 15 g, Magnoliae Officinalis Cortex (Houpo) 15 g, Raphani Semen (Laifuzi) 20 g, Atractylodis Macrocephalae Rhizoma (Baizhu) 40 g, Cannabis Semen (Huomaren) 30 g. | 2 months | Heal | (Cui and Wang 2008) |
| 4 | Antral gastritis | 102 cases, 48 males and 54 females, aged 18 to 87 years, with a duration of 1 week to 10 years. | **CR 10g, CP 10g,** Pinelliae Rhizoma Praeparatum (Zhibanxia) 10 g, Scutellariae Radix (Huangqin) 10 g, Coptidis Rhizoma (Huanglian) 3 g, Taraxaci Herba (Pugongying) 30 g, Zingiberis Rhizoma (Ganjiang) 5 g, Codonopsis Radix (Dangshen) 15 g, Glycyrrhizae Radix et Rhizoma (Gancao) 5 g, Trichosanthis Pericarpium (Gualoupi) 20 g, Salviae Miltiorrhizae Radix et Rhizoma (Danshen) 15 g. | 15 to 45 days | Total effective rate: 94.1%  Cure rate: 56.9% | (Chen 2009) |
| 5 | Irritable bowel syndrome | 68 cases, 26 males and 42 females, aged 22 to 68 years, with a duration of 1 to 6 years. | **CR 15g, CP 12g,** Atractylodis Macrocephalae Rhizoma (Baizhu) 15 g, Coicis Semen Praeparatum (Chaoyimi) 15 g, Euryales Semen (Qianshi) 15 g, CX 15 g, CH 12 g, Paeoniae Radix Alba (Baishao) 12 g, Saposhnikoviae Radix (Fangfeng) 10 g, Poria (Fuling) 20 g, Polygoni Multiflori Caulis (Shouwuteng) 30 g, Portulacae Herba (Machixian) 30 g, Glycyrrhizae Radix et Rhizoma (Gancao) 6 g. | 20 days | Total effective rate: 97% | (Yu 2011) |

**3.5. CR–CZ herb pair**

CZ is the dried rhizome of *Atractylodes lancea* (Thunb.) DC. or *Atractylodes chinensis* (DC.) Koidz which is classified within the family Asteraceae (Li et al. 2004) (**Figure S1**). CZ's chemical constituents identified as being of primary importance are sesquiterpenoids, triterpenoids, aromatic glycosides, *etc.* (Deng et al. 2016). Moreover, it has been demonstrated to possess various pharmacological effects, including hypoglycemic, antibacterial, anti-inflammatory, sedative, analgesic, and antitumor properties (Deng et al. 2016; Zhao 2009) (specifically, atractylol extract has been observed to inhibit cholangiocarcinoma in hamsters (Plengsuriyakarn et al. 2015)). The clinical formula containing CR- herb pair is known as Liu Yu Tang (LYT), which is frequently utilized to treat liver depression syndrome, defined as obstructions of substances such as qi, blood, or phlegm. The Cang Fu Dao Tan Pill (CFDTP) is a formula that is frequently employed in the field of gynaecology.


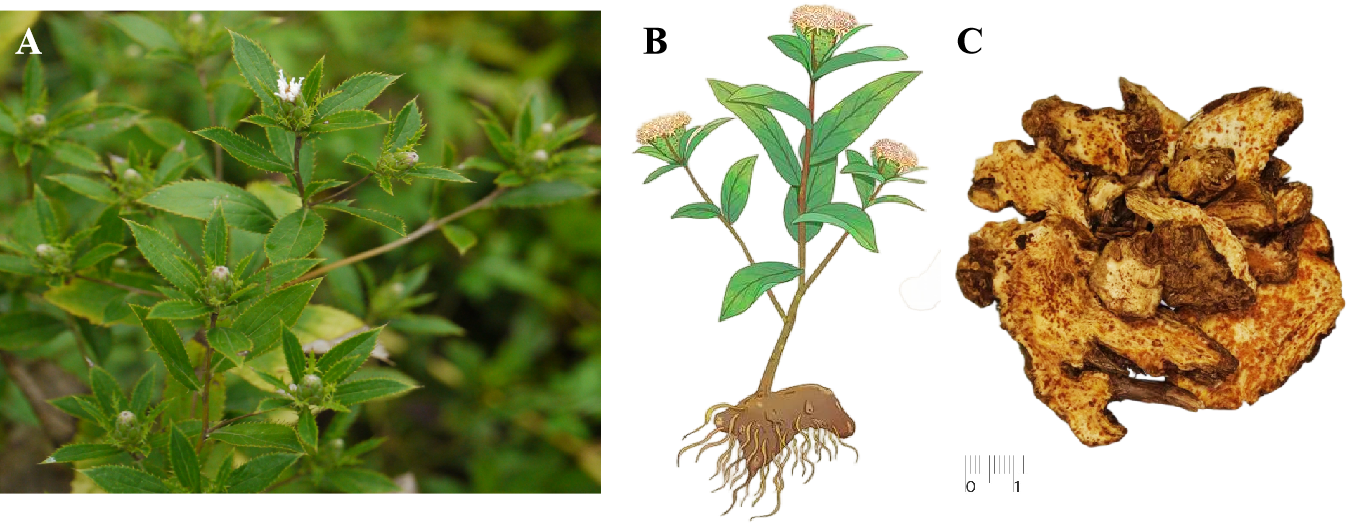


**FIGURE S1** **|** Schematic diagram of CZ plant. (A) The wild growing state of CZ plant, (B) Hand-painted whole-plant image, (C) CZ in the form of TCM decoction pieces

**3.5.1. Bioactive components variation of the CR-CZ herb pair**

Cao *et al.* utilized ultrahigh-phase liquid-quadrupole-time-of-flight mass spectrometry (UPLC-Q-TOF-MS/MS) to assay the chemical composition of CR-CZ herb pair alcohol extract. A total of 54 compounds were detected in this analysis, among which CR contained 20 compounds and CZ contained 41 compounds, and 7 of them were shared by both. Furthermore, the content of luteolin, atractylide Ⅰ, atractylide Ⅱ, atractylide Ⅲ, α-cyperone, and atractylodin in the alcoholic CR-CZ herb pair extract was also detected using the HPLC method. The average content of each component was, respectively: 0.0094, 0.0560, 0.0326, 3.600, 0.3967, 0.9357. (Cao et al. 2023)

Pharmacokinetic profiles of three active ingredients, atractylide Ⅰ, atractylide Ⅱ, and atractylide Ⅲ, in rats after gavage of CR-CZ herb pair extracts were examined by liquid chromatography-tandem mass spectrometry (LC-MS/MS). A subsequent examination of the concentration of the relevant active ingredient in rats revealed that atractylide Ⅲ was found to be much more abundant than atractylide Ⅰ *in vivo* (Cao et al. 2023). This phenomenon may be attributed to the occurrence of one-phase metabolism into the liver (Zhou et al., 2023), leading to structural alterations that facilitate the accumulation of atractylide Ⅲ. Alternatively, this discrepancy may be attributed to the reduced utilization of atractylide Ⅰ by the organism. The pharmacokinetic results demonstrated that atractylide Ⅰ, atractylide Ⅱ, atractylide Ⅲ (all fat-soluble substances) exhibited two absorption peaks (approximately 6 hours apart) after administration (Wan et al. 2021). These findings may be attributed to the following reasons: the herb enters the body in a molecular state in the epithelium of the stomach or small intestine, where the pH is low and conducive to the absorption of fat-soluble substances, resulting in the herb being passively transported into the bloodstream to form the first peak (Yuan et al. 2020). When the herb reaches the site close to the colon, the intestinal flora converts the drug to the glycoside form, and then the glycoside glucuronidation is absorbed to form a second peak. Alternatively, the phenomenon is related to the hepatic-intestinal cycle, in which glucuronidation occurs after the herb enters the liver and is excreted via the bile into the small intestine, where hydrolysis back to the prototype occurs, at which point the prototype herb is reabsorbed by the small intestine, resulting in a second peak (Durník et al. 2022) (**Table S5**).

**TABLE S5** **|** The main chemical composition of CR-CZ herb pair

| **NO.** | | **Name** | **2D Structure** | **Molecular Weight** | **Molecular**  **Formula** | **CAS** |
| --- | --- | --- | --- | --- | --- | --- |
| 1 | luteolin | | **** | 286.240 | C_15_H_10_O_6_ | 491-70-3 |
| 2 | atractylide Ⅰ | | **** | 230.000 | C_1__5_H_18_O_2_ | 73069-13-3 |
| 3 | atractylide Ⅱ | | **** | 232.000 | C_15_H_20_O_2_ | 73069-14-4 |
| 4 | atractylide Ⅲ | | **** | 248.320 | C_15_H_20_O_3_ | 73030-71-4 |
| 6 | atractylodin | | **** | 182.218 | C_13_H_10_O | 55290-63-6 |

**3.5.2. Compatibility effects of the CR-CZ herb pair**

Through network pharmacology, it was found that the main active ingredients of CR-CZ herb pair for the treatment of polycystic ovary syndrome (PCOS) are quercetin, digicitrine, kaempferol, and baicalein, all of which can inhibit the expression of inflammatory cytokines and chemokines, thus suppressing the inflammatory response and improving PCOS. And through PI3K-Akt signaling pathway, AGE-RAGE signaling pathway, Diabetic Complications, Hypoxia Inducible Factor-1 signaling pathway (HIF-1), FoxO signaling pathway, Interleukin-17 (IL-17) and other signaling pathways to regulate the corresponding core genes such as Interleukin-6 (IL-6), Akt Serine/Threonine Kinase (AKT1), Vascular Endothelial Growth Factor A (VEGFA), Epidermal Growth Factor (EGF), *etc*. Thereby regulating metabolism, promoting follicular development and ovulation, inhibiting inflammatory factors, reducing androgen production, *etc*., to achieve multi-target, multi-pathway treatment of PCOS ( Zheng and Zhao 2022).

**3.5.3. Clinical applications of CR-CZ herb pair**

CR-CZ herb pair is used more often to treat diseases affecting female patients. A comprehensive review of the extant literature on randomized controlled trials of CFDTP for the treatment of PCOS was conducted, and a meta-analysis was performed. The results indicated that CFDTP in combination with conventional western drug regimens is more efficacious than western drug therapy alone, it significantly increased the overall effective rate, ovulation rate, and pregnancy rate (*P*<0.01), reduced luteinizing hormone in serum hormone water bottles (*P*<0.01), body mass index, sign score (acne), TCM symptom score and ovarian volume (*P*<0.01) (Zhou et al. 2022). It can also treat phlegm stagnation-type amenorrhea (Wang et al. 2021) (**Table S6**).

**TABLE S6 |** Clinical application of CR-CZ herb pair

| **NO.** | **Clinical data** | | | | | **Ref.** |
| --- | --- | --- | --- | --- | --- | --- |
|  | **Diseases** | **Cases** | **Medicinal** | **Dosing cycles** | **Results** |  |
| 1 | Chronic non-atrophic gastritis | 60 cases, 34 males, 26 females, age (45.6±12.8) years, disease duration (6.3±3.9) years | **CR 9g, CZ 12g,** Magnoliae Officinalis Cortex (Houpo) 9g, CP 9g, Amomi Fructus (Sharen) 6g, Glycyrrhizae Radix et Rhizoma (Gancao) 6 g | 4 weeks | Total effective rate: 88.33% | (Yang 2019) |
| 2 | Fatty liver | 59 cases, 41 males and 18 females; mean age 42 years | **CR 10g, CZ 15g,** Massa Medicata Fermentata (Shenqu) 20 g, Gardeniae Fructus (Zhizi) 10 g,  CX 20 g | 2 months | Total effective rate: 84.75% | (Lei 2003) |
| 3 | Cyclomastopathy | 60 cases were female, aged 24 to 52 years, with a mean of 35 years. The duration of the disease ranged from 3 months to 11 years, with an average of 19 months. | **CR 15g, CZ 10g,** CX 12 g, Gardeniae Fructus (Zhizi) 12 g, Citri Reticulatae Pericarpium Viride (Qingpi) 10 g, Curcumae Radix (Yujin) 12 g, Gentianae Radix (Longdancao) 12 g, Prunellae Spica (Xiakucao) 12 g, Trichosanthis Fructus (Gualou) 15 g, Pinelliae Rhizoma Praeparatum (Banxia) 10 g, Arisaematis Rhizoma Praeparatum (Dannanxing) 10 g, Cremastrae Pseudobulbus (Shancigu) 10 g, Fritillariae Thunbergii Bulbus (Zhebeimu) 10 g, Gleditsiae Spina (Zaojiaoci) 10 g, Manis Squama (Chuanshanjia) 6 g, Citri Reticulatae Semen (Juhe) 10 g, Cynomorii Herba (Loulu) 10 g | 20 to 40 days | Total effective rate: 95.0%  Cure rate: 60.0% | (Liu and Chu 1999) |
| 4 | Amenorrhea | 56 cases, aged 16-45 years, with a duration of 4 months to 1 year. | **CR 15g, CZ 15g,** Poria (Fuling) 20 g, Pinelliae Rhizoma Praeparatum (Fabanxia) 15 g, CP 10 g, Arisaematis Rhizoma Praeparatum (Dannanxing) 10 g, Aurantii Fructus Praeparatus (Chaozhiqiao) 10 g, Massa Medicata Fermentata (Shenqu) 15 g, Glycyrrhizae Radix et Rhizoma (Gancao) 3 g, Astragali Radix (Huangqi) 30 g, Angelicae Sinensis Radix (Danggui) 15 g, CX 10 g, Gleditsiae Spina (Zaojiaoci) 15 g, Sappan Lignum (Sumu) 10 g, Coicis Semen (Yiyiren) 30 g, Spatholobi Caulis (Jixueteng) 15 g, Zingiberis Rhizoma Recens (Shengjiang) 3 slices | 6 months | Total effective rate: 92.86% | (Bao 2020) |
| 5 | Stein-leventhal syndrome | 42 cases, age 25-38 years, 27 to 36 years | **CR 9g, CZ 10g,** Persicae Semen (Taoren) 6 g, Angelicae Sinensis Radix (Danggui) 12 g, CX 10 g, Carthami Flos (Honghua) 6 g, Arisaematis Rhizoma Praeparatum (Dannanxing) 9 g, Poria (Fuling) 15 g, Pinelliae Rhizoma Praeparatum (Fabanxia) 10 g, CP 6 g, Aurantii Fructus (Zhiqiao) 6 g, Prunellae Spica (Xiakucao) 10 g, Zingiberis Rhizoma Recens (Shengjiang) 6 g, Glycyrrhizae Radix et Rhizoma (Gancao) 6 g | 64 days | Total effective rate: 88.1% | (Zhang 2013) |
| 6 | Dysfunctional uterine bleeding | 34 cases, 18 to 46 years old, duration of illness 2 months to 2 years | **CR 12g, CZ 9g,** Pinelliae Rhizoma (Banxia) 9 g, Poria (Yunling) 12 g, CP 6 g, Arisaematis Rhizoma (Nanxing) 6 g, Aurantii Fructus (Zhiqiao) 6 g, Zingiberis Rhizoma Recens (Shengjiang) 9 g, Cinnamomi Ramulus (Guizhi) 6 g, Moutan Cortex (Danpi) 9 g, Paeoniae Radix Rubra (Chishao) 6 g, Glycyrrhizae Radix et Rhizoma (Gancao) 12 g | 10 to 20 days | Total effective rate: 79% | (Wang 1996) |

A paucity of research exists on the changes in the efficacy of CR-CZ herb pair at relevant ratios. In clinical practice, CR-CZ is often applied in PCOS by regulating multiple pathways in a 9:10 ratio (Ji 2022). At a ratio of 2:1, it is commonly used for reflux esophagitis, which may be related to the decrease of IL-6 and TNF-α (Fan 2019). Additionally, CR-CZ herb pair has been the subject of comparatively limited pharmacology research, with the bulk of studies concentrating on network pharmacology. Consequently, there is a necessity for further investigation into its specific mechanism of action to ensure a comprehensive understanding of its pharmacodynamics.

**3.6. CR****-CH herb pair**

CH is the dried root of *Bupleurum chinense* DC. or *Bupleurum scorzonerifolium* Willd (**Figure S2**). It is used in the treatment of colds and fever, chest and abdominal distension and pain, menstrual disorders, uterine prolapse, and prolapse of the anus (Li et al. 2004). The principal active components of CH are saponins, volatile oils, flavonoids, and polysaccharides. CR and CH, as classic herb pair in clinical practice, is represented by CHSGP and Tongqi powder (TQP), which can be used to relieve liver qi constraint (Yan et al. 2018). Meanwhile, CR-CH herb pair is a classic combination commonly used clinically to treat depression in a ratio of 3:4 (Lin et al. 2024)or 1:1 (Gao et al. 2023).


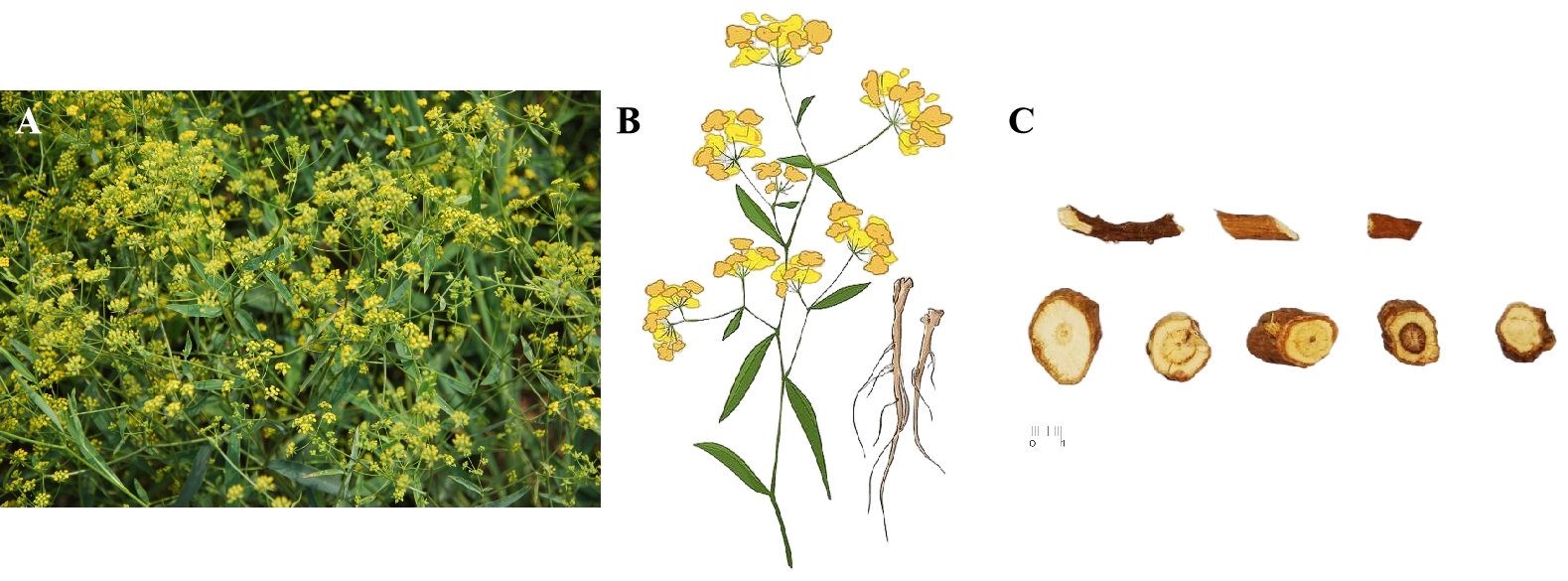


**FIGURE S2 |** Schematic diagram of CH plant. (A) The wild growing state of CH plant, (B) Hand-painted whole-plant image, (C) CH in the form of TCM decoction pieces

**3.6.1 Compatibility effects of the CR-CH herb pair**

**3.6.1.1. Anti-depressant effect**

Guo used the Gene Expression Omnibus data to analyze five core genes of CR-CH herb pair for depression: mitogen-activated protein kinase 14 (MAPK14), matrix metallopeptidase 9 (MMP9), epidermal growth factor (EGF), MYC proto-oncogene (MYC), and monoamine oxidase A (MAOA). It is worthy of further study (Guo et al. 2024).

**3.6.1.2. Effects on the central nervous system**

Lv *et al.* conducted a preliminary verification through network pharmacology, which indicated that CR-CH herb pair may treat epilepsy by signaling through pathways such as neuroactive ligand-receptor interactions, estrogen signaling pathways, and cholinergic synapses (Lv et al. 2023). Zhu *et al.* used network pharmacology to verify the mechanism of action of CR-CH herb pair in the treatment of Alzheimer's disease and epilepsy. The mechanism may be through the modulation of IL-17 signaling pathway, AGE-RAGE signaling pathway, and TNF pathway, *etc.*, which act on caspase 3 (CASP3), tumor protein 53 (TP53), vascular endothelial growth factor A (VEGFA), and estrogen receptor 1 (ESR1), IL6, and other targets may be effective in treating Alzheimer's disease and epilepsy (Zhu et al. 2023). However, these findings have yet to be substantiated through rigorous validation in animal and clinical trials, warranting further exploration in future research.

**3.6.1.3. Other Compatibility Effects**

Qing used multilevel data integration and molecular docking, quercetin, stigmasterol, isorhamnetin, and kaempferol were found to be the main active ingredients in the anti-hepatocellular carcinoma effect of CR-CH herb pair. CR-CH herb pair regulates the p53 signaling pathway, cellular senescence, cell cycle, and retinol metabolism and thus regulates hepatocellular carcinoma progression (**Figure S3**). The cyclin-dependent kinases 4 (CDK4), Checkpoint Kinase 1 (CHEK1), Cyclin B1 (CCNB1), and Cyclin-dependent kinase inhibitor 2A (CDKN2A) were identified as key targets of CR-CH herb pair in the treatment of hepatocellular carcinoma by The Cancer Genome Atlas (TCGA)-liver hepatocellular carcinoma (LIHC) database (Qing et al. 2022).


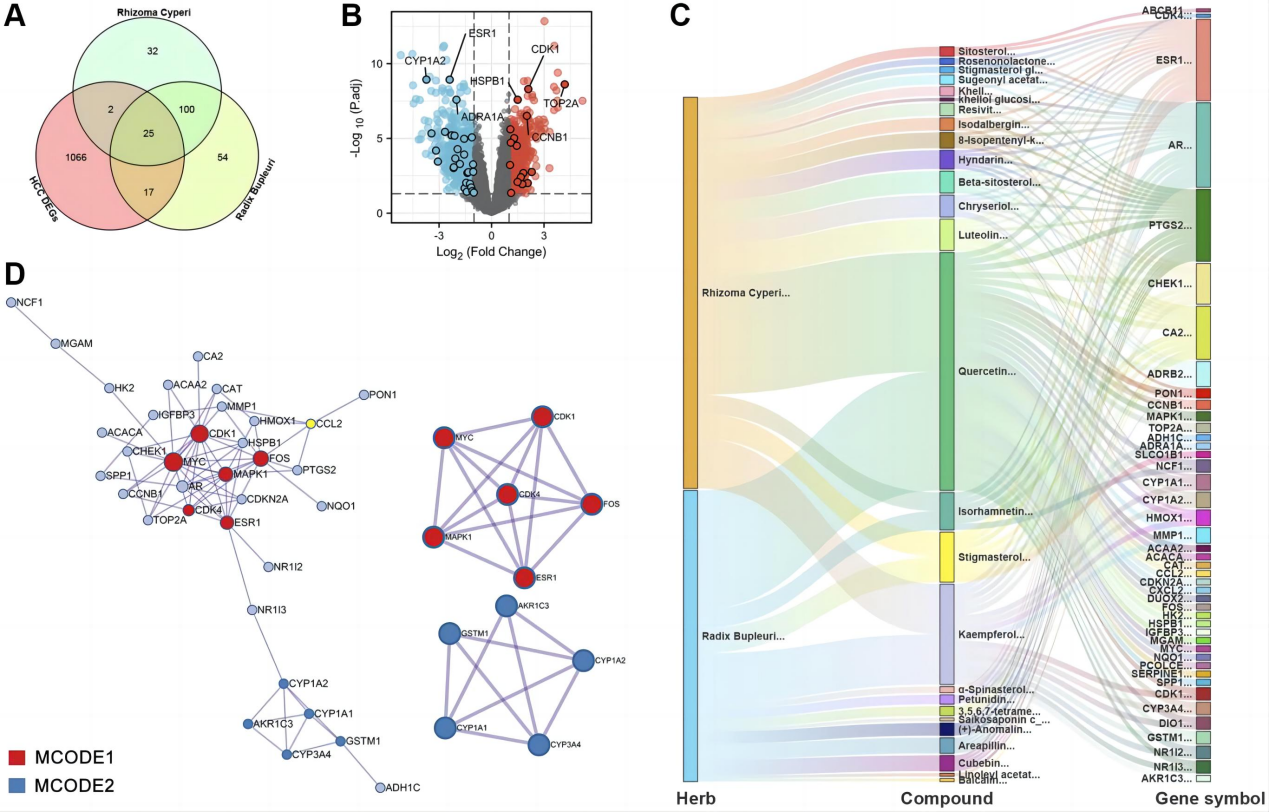


**FIGURE S3 |** PPI and H-C-T network analysis of 44 potential therapeutic targets for CXP in HCC(Qing et al. 2022). (A) Venn diagram. (B) The distribution of 44 potential therapeutic targets of CXP in the treatment of HCC in the volcano plot of DEGs in HCC. (C) H-C-T network analysis. (D) PPI network and gene clustering analysis.

**3.6.2. Clinical applications of** **CR-CH herb pair**

CR-CH herb pair is primarily utilized in clinical settings to treat digestive system diseases, including gastroesophageal reflux disease (GERD), chronic gastritis, bile reflux gastritis, and other related conditions. Chaihuxiangfu Fang (CHXFF,), with CR-CH herb pair as the primary active ingredient, has been utilized for the treatment of GERD, and the results showed that there were 36 cases with an overall effective rate of 94.4% (Su et al. 2016). CHXFF has also been utilized in the treatment of chronic gastritis. Following a designated period of treatment, patients have demonstrated notable improvements in their gastritis symptoms (Luo et al. 2015). Concurrently, CHXFF has been utilized in the treatment of bile reflux and gastritis. Patients were monitored for one year, with no reemergence of the condition observed following a two-month treatment duration (Su et al. 2015) (**Table S7**)

**TABLE S7 |** Clinical application of of CR-CH herb pair

| **NO.** | **Clinical data** | | | | | **Ref.** |
| --- | --- | --- | --- | --- | --- | --- |
|  | **Diseases** | **Cases** | **Medicinal** | **Dosing cycles** | **Results** |  |
| 1 | Gastric ulcer | 70 cases,48 males and 22 females, aged 19 - 68, with a history of gastric ulcer, a duration of 0.5 years to 12 years | **CR 10g, CH 10g,** Coptidis Rhizoma (Huanglian) 15g, Astragali Radix (Huangqi) 15g, Arcae Concha (Walengzi) 15g, Ostreae Concha (Muli) 15g, Notoginseng Radix et Rhizoma (Sanqi) 3g, Curcumae Radix (Yujin) 10g, Euodiae Fructus (Wuzhuyu) 5g, Euodiae Fructus (Renshen) 6g, Glycyrrhizae Radix et Rhizoma (Gancao) 6g | 4 weeks. | Total effective rate:94.3% | (Lin 2012) |
| 2 | Post-stroke depression | 40 cases, 22 males, 18 females, mean age (59.40±10.20) years, mean duration of disease (7.00±0.80) months, 12 cases of cerebral hemorrhage and 28 cases of cerebral infarction. | **CR 15g, CH 15g,** CP 12 g, CX 15 g, Aurantii Fructus (Zhiqiao) 12 g, Paeoniae Radix Alba (Baishao) 12 g, Ziziphi Spinosae Semen (Suanzaoren) 30 g, Polygalae Radix (Yuanzhi) 10 g, Draconis Os (Longgu) 30 g, Ostreae Concha (Muli) 30 g, Curcumae Radix (Yujin) 15 g, Angelicae Sinensis Radix (Danggui) 15 g, Salviae Miltiorrhizae Radix et Rhizoma (Danshen) 15 g, Moutan Cortex (Danpi) 10 g | 4 weeks | Total effective rate: 90.00% | (Liu and Zhong 2012) |
| 3 | Nonalcoholic Fatty Liver Disease (NAFLD) | 41 cases, 29 male, 12 female; age 21-68 years, mean (44.2±7.6) years; duration of disease 0.5-15 years. | **CR 9g, CH 12g,** CP 12 g, CX 9 g, Aurantii Fructus (Zhiqiao) 9 g, Paeoniae Radix Alba (Baishao) 9 g, Glycyrrhizae Radix et Rhizoma (Gancao)3 g | 3 months | Total effective rate: 90.2% | (Pan and Huang 2009) |
| 4 | Functional dyspepsia | 36 cases, 15 male, 21 female; age 26-60 years, mean (37.87±6.01) years; disease duration 7-48 months. | **CR 10g, CH 10g,** Paeoniae Radix Alba (Baishao) 15 g, Aurantii Fructus (Zhiqiao) 15 g, CX 15 g, Glycyrrhizae Radix et Rhizoma (Gancao) 6 g,Citri Sarcodactylis Fructus (Foshou) 10 g, Hordei Fructus Germinatus (Gumaiya) 15 g (Note: barley and wheat sprouts combined), CP 10 g, Pinelliae Rhizoma (Banxia) 10 g, Magnoliae Officinalis Cortex (Houpo) 10 g, Massa Medicata Fermentata (Shenqu) 10 g | 4 weeks | Total effective rate: 83.33% | (Jin et al. 2012) |
| 5 | Primary dysmenorrhea | 30 cases, 18 males, 12 females; age 30-75 years; duration of disease 2-16 years | **CR 10g, CH 10g,** Persicae Semen (Taoren) 10 g, Angelicae Sinensis Radix (Danggui) 10 g, CX 10 g, Linderae Radix (Wuyao) 10 g, Curcumae Radix (Yujin) 10 g, Achyranthis Bidentatae Radix (Niuxi) 10 g, Rehmanniae Radix Praeparata (Shudihuang) 12 g, Paeoniae Radix Alba (Baishao) 15 g, Corydalis Rhizoma (Yanhusuo) 15 g, Carthami Flos (Honghua) 6 g, Glycyrrhizae Radix et Rhizoma (Gancao) 6 g | 3 months | Total effective rate: 93.75% | (Hou and Guo 2009) |
| 6 | Mammary hyperplasia | Female,35 years old. | **CR 15g, CH 15g,** Angelicae Sinensis Radix (Danggui) 15 g, Paeoniae Radix Alba (Baishao) 15 g, Asparagi Radix (Tiandong) 15 g, Curcumae Radix (Yujin) 15 g,Citri Reticulatae Pericarpium Viride (Qingpi) 15 g, Citri Reticulatae Semen (Juhe) 15 g, Epimedii Folium (Yinyanghuo) 12 g, Cremastrae Pseudobulbus Pleiones Pseudobulbus (Shancigu) 15 g, Hordei Fructus Germinatus (Maiya) 20 g, Scutellariae Barbatae Herba (Banzhilian) 20 g, Olibanum (Ruxiang) 10 g, Myrrha (Moyao) 10 g Poria (Fuling) 15 g, Laminariae Thallus Eckloniae Thallus (Kunbu) 15 g, Ostreae Concha (Muli) 15 g Corydalis Rhizoma (Yuanhu) 20 g, Glycyrrhizae Radix et Rhizoma (Gancao) 6 g. | 1 month | Heal | (Qian 2012) |
| 7 | Gastroesophageal reflux disease | Female, 42 years old. | **CR 25g, CH 15g,** Coptidis Rhizoma (Huanglian) 6 g, Pinelliae Rhizoma (Banxia) 25 g, Poria (Fuling) 20 g, Atractylodis Macrocephalae Rhizoma Praeparatum (Chaobaizhu) 25 g, Eriobotryae Folium (Pipaye) 15 g, Corydalis Rhizoma (Yanhusuo) 25 g, Toosendan Fructus (Chuanlianzi) 15 g, Aurantii Fructus Immaturus (Zhishi) 30 g, Bambusae Caulis In Taenias (Zhuru) 15 g, CP 12 g, Euodiae Fructus (Wuzhuyu) 6 g, Glycyrrhizae Radix et Rhizoma Praeparata cum Melle (Gancao) 6 g | - | Heal | (Su et al. 2016) |
| 8 | Bile reflux gastritis | Female,34 years old. | **CR 15g, CH 15g,** Perillae Caulis (Sugeng) 20 g, Aurantii Fructus (Zhiqiao) 15 g, CP 15 g, Crataegi Fructus Torrefactus (Jiaoshanzha) 20 g, Hordei Fructus Germinatus Torrefactus (Jiaomaiya) 20 g, Massa Medicata Fermentata (Shenqu) 20 g, Gardeniae Fructus (Jhizi) 15 g, Taraxaci Herba (Pugongying) 20 g, Forsythiae Fructus (Lianqiao) 15 g, Codonopsis Radix (Dangshen) 20 g, Atractylodis Macrocephalae Rhizoma (Baizhu) 20 g, Dioscoreae Rhizoma (Shanyao) 25 g, Bletillae Rhizoma (Baiji) 20 g, Galli Gigerii Endothelium Corneum (Jineijin) 20 g | 30 doses | Heal | (Pan 2009) |

“-” indicates that it was not mentioned in the original reference.

The primary application of CR-CH herb pair is in the treatment of central nervous system diseases, which has led to the treatment and improvement of depression, epilepsy, and other related conditions. While bioinformatic approaches have identified candidate protein targets and pathways mediating the CR-CH herb pair's effects on central nervous system diseases, *in vivo* experimental validation using animal models is still warranted. Furthermore, CR-CH herb pair has shown clinical efficacy in the treatment of digestive system diseases and liver cancer. However, at present, there are very few studies on the chemical compositions of CR-CH herb pair. Most of the studies focus on the chemical composition of CR-CH herb pair in complex prescriptions, lacking the study of the chemical composition of these 2 drugs before and after compounding. It is difficult to link the pharmacological effects and mechanisms of action to the material basisepilepsy.

**References:**

Bao Y. (2020). Modified Cangfu Daotan Pills in the treatment of 56 cases of phlegm-dampness blocking amenorrhea. *Journal of Practical Traditional Chinese Medicine, 36*(4), 437-438.

Cao Y., Dai G. L., Li F. R., *et al*. (2023). Determination of atractylenolide Ⅰ, Ⅱ and Ⅲ in rat plasma by LC-MS/MS and study on pharmacokinetics of Atractylodes-Cyperi rhizoma extract in Rats. *Chinese Pharmaceutical Journal, 58*(17), 1582-1588.

Cao Y., Dai G. L., Wang Y. Q., *et al*. (2023). Analysis and evaluation on Atractylodes-Cyperus alcohol extract by UPLC-Q-TOF-MS/MS and HPLC. *Chinese Pharmaceutical Journal, 58*(19), 1736-1743.

Chen, H. L. (2009). Banxia Xiexin Decoction in the Treatment of 102 Cases of Antral Gastritis. *Journal of Practical Traditional Chinese Medicine*, *25*(9), 600-601.

Chen, Q., Yin, L. H., Mao, Y., Bu, X. C. (2020). Clinical effect of Jiawei Yueju pills in treatment of prediabetes: An analysis of 30 cases. *Hunan Journal of Traditional Chinese Medicine*, *36*(6), 9-11.

Cheng, J. N., Shi, Y. A., and Liu, S. Q. (1995). Clinical Observation on 111 Cases of Upper Digestive Tract Ulcer Treated with Yueju Pill. Inner Mongolia Journal of Traditional Chinese Medicine (2), 7.

Cui, H. Y., Cui, J. N., Wang, Q. G. (2009). Clinical application of Yueju pill in gynecological menstrual disease. *Journal of Sichuan of Traditional Chinese*, *27*(2), 93-94.

Cui, Q. Z., and Wang, X. Y. (2008). Summary of 54 Cases of Constipation Treated by Chaihu Shugan Powder. Guiding Journal of Traditional Chinese Medicine and Pharmacy (4), 25+38.

Deng A. P., Li Y., Wu Z. T., *et al*. (2016). Advances in studies on chemical compositions of Atractylodes lancea and their biological activities. *China Journal of Chinese Materia Medica, 41*(21), 3904-3913.

Durník R., Šindlerová L., Babica P., *et al*. (2022). Bile acids transporters of enterohepatic circulation for targeted drug delivery. *Molecules, 27*(9), 2961.

Fan S. J., Hou J., Huang Y. W. (2019). Effect of Liuyu Decoction on clinical efficacy of reflux esophagitis and its effects on TNF-α and Il-6 in patients. *Chinese Archives of Traditional Chinese Medicine, 37*(1), 200-202.

Gao X. S., Li L., Wang A. N., *et al*. (2023). Effects of simplified Chaihu Shugan Powder on behaviors and regulating the metabolism of tryptophan in raphe nucleus of depressive rats. *Laboratory Animal Science, 40*(1), 16-21.

Gu, X. D. (2008). Clinical Observation on 60 Cases of Chronic Gastritis Treated with Modified Liangfu Pills Western. *Journal of Traditional Chinese Medicine* (11), 45.

Guo, F., and He, B. Y. (2011). Aifu Nuangong Decoction in the treatment of 91 cases of dysmenorrhea. *Journal of Emergency in Traditional Chinese Medicine*, *20*(9), 1474.

Guo Z. Y., Zeng Y., Long Q., *et al*. (2024). Exploring the mechanism of action of bupleurum-cyperus rotundus in treating depression based on network pharmacology and molecular docking techniques. *Journal of International Psychiatry, 51*(1), 78-85+90.

Hou X. X. and Guo J. H. (2009). Taohong Siwu Decoction in the treatment of 32 cases of primary dysmenorrhea. *Guangming Journal of Chinese Medicine, 24*(11), 2138.

Hu, T. (2012). 58 Cases of Acute Jaundice Hepatitis Treated with Yueju Pills. *Chinese Medicine Modern Distance Education of China*, *10*(18), 15.

Ji S. L., Lu S. J., Zheng L., *et al*. (2022). A study on network pharmacological mechanism of Cangzhu-Xiangfu in treating polycystic ovary syndrome. *Clinical Journal of Chinese Medicine, 14*(32), 109-114.

Ji S. L., Lu S. J., Zheng L., *et al*. (2022). A study on network pharmacological mechanism of Cangzhu-Xiangfu in treating polycystic ovary syndrome. *Clinical Journal of Chinese Medicine, 14*(32), 109-114.

Jia, R., Jia, D. Q., Jia, D. W. (1996). Jiawei Liangfu Pill in the treatment of 58 cases of chronic cholecystitis. *Inner Mongolia Journal of Traditional Chinese Medicine*(S1), 102.

Jin L. N., Gan C., He L. H. (2012). Chaihu Shugan Powder in the treatment of functional dyspepsia liver stomach disharmony syndrome 36 cases. *Jiangxi Journal of Traditional Chinese Medicine, 43*(1), 19-20.

Lei Q. S. (2003). Treating 59 cases of fatty liver with yueju pill. *Henan Traditional Chinese Medicine* (3), 55.

Li, F. P., and Liu, X. G. (2015). Observation on the curative effect of 44 cases of benign prostatic hyperplasia of deficiency cold and blood stasis type treated by internal and external application of Aifu Nuangong pill. *New Chinese Medicine*, *47*(6), 113-114.

Li, M. (2022). Dingxiang Shidi Powder combined with Yueju Pill in the treatment of 30 cases of refractory gastroesophageal reflux disease of liver-stomach disharmony type. *Zhejiang Journal of Traditional Chinese Medicine*, *57*(10), 739.

Li, S. D., and Liu, F. Y. (1985). Clinical observation on treating 40 cases of bile reflux gastritis with Jiawei Liangfu Pills. *Liaoning Journal of Traditional Chinese Medicine* (7), 36.

Li X. L., Eishi Y., Bai Y. Q., *et al*. (2004). Expression of the SRY-related HMG box protein SOX2 in human gastric carcinoma. *International journal of oncology, 24*(2), 257-263.

Li, Y. S., Jiang, Y. P., Yang, Z., and Liu, C. (2015). Sanhe Decoction in the treatment of 30 cases of senile chronic gastritis of cold coagulation and qi stagnation with blood stasis type. *Jiangxi Journal of Traditional Chinese Medicine*, *46*(4), 44-45.

Li, Z. X., and He, J. (2013). Aifu Nuangong Pills combined with Dingkun Dan in the treatment of 63 cases of dysfunctional uterine bleeding. *Inner Mongolia Journal of Traditional Chinese Medicine*, *32*(1), 9.

Lin J. Y. (2012). Modified Zuojin Decoction in the treatment of 70 cases of gastric ulcer. *Shaanxi Journal of Traditional Chinese Medicine, 33*(11), 1484-1485.

Lin J. Y., Duan Y., Long M. Q., *et al*. (2024). Pharmacodynamic substances and action mechanisms of Chaihu Shugansan in antidepressant treatment： A review. *Chinese Journal of Experimental Traditional Medical Formulae, 30*(10), 246-257.

Liu J. and Zhong C. (2012). Chaihu Shugan Powder in the treatment of post-stroke depression 40 cases. *Journal of Emergency in Traditional Chinese Medicine, 21*(5), 788.

Liu S. Z. and Chu M. J. (1999). Modified Yueju Pills in the treatment of 60 cases of hyperplasia of mammary glands. *Western Journal of Traditional Chinese Medicine*(5), 41-42.

Liu, X. T., & Yan, Q. H. (2013). Clinical study on 60 cases of alcoholic fatty liver ( qi stagnation and phlegm dampness type ) treated with Yueju Pill. *Guide of China Medicine*, 11(10), 670-671.

Lu, H. F. (2015). Clinical observation on treating 98 cases of functional dyspepsia with self-made Ningwei Jieyu decoction. *Chinese Journal of Traditional Medical Science and Technology, 22*(3), 283.

Luo M. X., Li L. X., Wei C. L., *et al*. (2015). The treatment of chronic gastritis Li Pei Bupleurum Cyperi party. *Journal of Practical Traditional Chinese Internal Medicine, 29*(12), 12-13.

Lv T. T., Lan S. H., Li R. N., *et al*. (2023). The mechanism of Chaihu combined with Xiangfu in the treatment of epilepsy based on network pharmacology. *Western Journal of Traditional Chinese Medicine, 36*(5), 1-6.

Mou, M. O. (2009). Modified Yueju Pill in the treatment of 50 cases of gastric neurosis. *Zhejiang Journal of Traditional Chinese Medicine*, *44*(9), 675.

Pan F. M. and Huang J. R. (2009). Clinical observation on 82 cases of nonalcoholic fatty liver treated with Chaihushugansan. *Journal of Li-shizhen Traditional Chinese Medicine, 20*(8), 2010-2011.

Plengsuriyakarn T., Matsuda N., Karbwang J., *et al*. (2015). Anticancer activity of Atractylodes lancea (Thunb.) DC in a hamster model and application of PET-CT for early detection and monitoring progression of cholangiocarcinoma. *Asian Pacific Journal of Cancer Prevention, 16*(15), 6279-6284.

Qi, X. H. (2014). Yueju Pills combined with Simo Decoction in the treatment of gastrointestinal dysfunction in 35 cases. *Zhejiang Journal of Traditional Chinese Medicine*, *49*(3), 181.

Qian Y. P. (2012). Rupi Decoction in the treatment of 112 cases of hyperplasia of mammary glands. *Chinese Journal of Ethnomedicine and Ethnopharmacy, 21*(1), 104.

Qing L., Pan B., He Y., *et al*. (2022). Exploring the mechanisms underlying the therapeutic effect of the Radix Bupleuri-Rhizoma Cyperi herb pair on hepatocellular carcinoma using multilevel data integration and molecular docking. *Aging (Albany NY), 14*(22), 9103-9127.

Ren, Y. H. (2010). 50 Cases of Pharyngeal Paraesthesia Treated with Modified Yueju Pill. *Traditional Chinese Medicinal Research*, *23*(6), 52-53.

Shen, Z. L. (2007). Clinical Observation on 30 Cases of Angina of Coronary Artery Disease Treated by Modified Yuejuwan. *Hunan Journal of Traditional Chinese Medicine* (3), 11-13.

Shi, C. (2005). Treatment of 60 Cases of Functional Hypopepsia with Yueju Pill and Its Modification. *Modern Chinese Clinical Medicine* (1), 15-17.

Su H. F., Qin W. Y., Wang X. P., *et al*. (2015). Professor Li Pei 's experience in treating bile reflux gastritis with Chaihu Xiangfu Decoction. *Inner Mongolia Journal of Traditional Chinese Medicine, 34*(7), 47.

Su H. F., Wang L. M., Chen X. L., *et al*. (2016). Clinical observation on Chaihu Xiangfu prescription in the treatment of gastroesophageal reflux disease for 36 cases. *Guangming Journal of Chinese Medicine, 31*(7), 959-961.

Sun, S. (2008). Clinical Observation on Treatment of 44 Cases of Hyperlipidemia with Modified“Yueju Pill”. *Shanghai Journal of Traditional Chinese Medicine* (1), 35-36.

Tong, Z. H., Xu, Z. C., Feng, B., Jin, W. D., Wang, H. Q., Shen, Y., & Chen, H. (2007). Modified Yueju Pills in the treatment of 46 cases of hyperprolactinemia caused by antipsychotics. *Zhejiang Journal of Traditional Chinese Medicine* (1), 31.

Wan Y., Shen Y. M., Zou J. F., *et al*. (2021). The intestinal absorption characteristics of five active components in Lizhong Decoction. *Acta Pharmaceutica Sinica, 56*(6), 1689-1695.

Wang, G. F., Sun, S. D., Liu, S. J., Li, X., & Han, H. X. (2017). Clinical Study on Yuejuwan Decotion for 36 Patients with Hepatitis B. *Chinese Journal of Medicinal Guide*, *19*(4), 388+390.

Wang J. F. (1996). Cangfu Daotan Pills combined with Guizhi Fuling Pills in the treatment of 34 cases of functional uterine bleeding. *Jiangxi Journal of Traditional Chinese Medicine* (5), 33.

Wang, L., & Zhao, S. J. (2000). Clinical Observation on Chronic Gastritis Treated with Modified Yueju Pills,a Report of 60 Cases. *Shanxi Journal of Traditional Chinese Medicine* (4), 11-12.

Wang, Q. L. (2010). Modified Yueju Pill in the Treatment of Neurogenic Headache. *Shanxi Journal of Traditional Chinese Medicine*, *26*(10), 35.

Wang, X. L. (2015). Observation on the curative effect of Aifu Nuangong Pill in the treatment of 29 cases of dysmenorrhea of cold coagulation and qi stagnation and blood stasis type. *Journal of Gansu University of Chinese Medicine*, *32*(3), 38-39.

Wang, X. N., & Yi, Y. (2023). Efficacy observation of Yueju pill and Danshen Yin for treating coronary heart disease caused stable angina pectoris complicated with anxiety by syndrome differentiation. *Shanxi Journal of Traditional Chinese Medicine*, *39*(4), 9-11.

Wang Y., Zhang M. M., Jiang H. X., et al. (2021). Application of drug pairs in amenorrhea. *Henan Traditional Chinese Medicine, 41*(11), 1639-1642.

Yan M. L., Yang L., Hou A. J., *et al*. (2018). Research progress on chemical composition and pharmacological effect of Bupleurum chinense. *Information on Traditional Chinese Medicine, 35*(5), 103-109.

Yang, D. D., & Yan, H. C. (2013). Chaihu Shugan Powder in the treatment of 47 cases of hyperplasia of mammary glands. *Journal of Practical Traditional Chinese Medicine*, *29*(10), 822.

Yang X. S. (2019). Clinical observation of Xiangsha Pingwei Powder in the treatment of 60 cases of chronic non-atrophic gastritis. *Guide of China Medicine, 17*(29), 189-190.

Yu, S. Y., & Yu, R. J. (2011). Tengchaifu Decoction in the treatment of 68 cases of irritable bowel syndrome of liver depression and spleen deficiency type. *Shaanxi Journal of Traditional Chinese Medicine*, *32*(5), 535-536.

Yuan J., Wei F., Luo X., *et al*. (2020). Multi-component comparative pharmacokinetics in rats after oral administration of Fructus aurantii extract, naringin, neohesperidin, and naringin-neohesperidin. *Frontiers in pharmacology, 11*, 933.

Zhang, M., & Xie, Q. Y. (2015). Clinical Observation on 30 Cases of Pre-diabetes Treated with Modified Yueju Pill. *Zhejiang Journal of Traditional Chinese Medicine*, *50*(9), 668.

Zhang W. (2013). Clinical observation on treating 42 cases of polycystic ovary syndrome with Cangfu Daotan Pill. *Guiding Journal of Traditional Chinese Medicine and Pharmacy, 19*(11), 101-102.

Zhang, Y., Feng, Y. H., Zhang, H., Wang, X. J., Sun, Y. P., Li, N., & Zhang, T. (2011). 60 Cases of Chronic Pharyngitis Treated with Yueju Pill. *Chinese Journal of Experimental Traditional Medical Formulae*, *17*(10), 297.

Zhao A. M. (2009). Study on pharmacological effects of Rhizoma Atractylodis. *Guangming Journal of Chinese Medicine, 24*(1), 181-182.

Zhou, Q. S., & Xiang, S. Y. (2012). 56 cases of chronic superficial gastritis were treated with Sini Powder and Liangfu Pill. *Journal of Practical Traditional Chinese Medicine*, *28*(5), 371.

Zhou S. N., Wu S. H., Sun Z., *et al*. (2023). Metabolites and metabolic pathway analysis of atractylenolide I in rats based on UHPLC-Q-Orbitrap HRMS. *Journal of Shenyang Pharmaceutical University, 40*(11), 1439-1448+1557.

Zhou T. Y., Sui J., Meng Y. X., *et al*. (2022). Systematic evaluation and trial sequential analysis of the clinical efficacy of Cangfu Daotan Pills in the treatment of polycystic ovary syndrome. *Journal of Li-shizhen Traditional Chinese Medicine, 33*(5), 1235-1241.

Zhu X. M., Chen W., Hong H. Z., *et al*. (2023). The study of alzheimer disease and epilepsy of "Chaihu-Xiangfu" drugs on alzheimer's disease based on network pharmacology and molecular docking technology. *Journal of Practical Traditional Chinese Internal Medicine, 37*(11), 8-13+144-147.
